# Entropy Fluctuations and Correlations in Compressible Turbulent Plane Channel Flow

**DOI:** 10.3390/e26060530

**Published:** 2024-06-20

**Authors:** G. A. Gerolymos, I. Vallet

**Affiliations:** Faculty of Science and Engineering, Sorbonne Université, 4 Place Jussieu, 75005 Paris, France

**Keywords:** turbulent boundary-layers, compressible boundary-layer, turbulence theory

## Abstract

The thermodynamic turbulence structure of compressible aerodynamic flows is often characterised by the correlation coefficient of entropy with pressure or temperature. We study entropy fluctuations $s^{\prime }$ and their correlations with the fluctuations of the other thermodynamic variables in compressible turbulent plane channel flow using dns data. We investigate the influence of the hcb (Huang–Coleman–Bradshaw) friction Reynolds number ($100\;\;⪅\;\;Re_{\tau^{★}}\;\;⪅\;1000\;$) and of the centreline Mach number ($0.3\;\;⪅\;\;{\bar{M}}_{{\mathrm{CL}}_{x}}\;\;⪅\;\;2.5$) on the magnitude and location of the peak of the root-mean-square $s_{\mathrm{rms}}^{\prime }$. The complete series expansions of $s^{\prime }$ with respect to the fluctuations of the basic thermodynamic variables (pressure *p*, density ρ and temperature *T*) are calculated for the general case of variable heat-capacity $c_{p}\left(T\right)$ thermodynamics. The correlation coefficients of $s^{\prime }$ with the fluctuations of the basic thermodynamic quantities ($c_{s^{\prime }p^{\prime }}$, $c_{s^{\prime }\rho^{\prime }}$, $c_{s^{\prime }T^{\prime }}$), for varying $\left(Re_{\tau^{★}},{\bar{M}}_{{\mathrm{CL}}_{x}}\right)$, are studied. Insight on these correlations is provided by considering the probability density function (pdf) of s′ and its joint pdfs with the other thermodynamic variables.

## 1. Introduction

Although compressible turbulence is inherently connected with the interaction of the fluctuations of the thermodynamic variables with the velocity field, thermodynamic variables have their own transport equations describing their interaction with the velocity field and with one another. The transport equations for pressure *p*, temperature *T* and enthalpy *h*, are derived [1] (p. 709), using thermodynamic derivatives, from two generating equations, continuity and entropy production. Written in nonconservative form, continuity $D_{t}\rho \;\;=\;\;-\rho \Theta$ (where $D_{t}$ is the substantial derivative) introduces the effect of dilatation $\Theta \;\;:=\;\partial_{x_{\ell }}u_{\ell }$ on the dynamics of thermodynamic variables, whereas entropy production $\rho D_{t}s\;\;=\;\;\left(\tau \;:\;S-\mathrm{div}\vec{q}\right)/T$ introduces the dissipative effects (τ is the molecular viscous stress tensor, S is the strain rate tensor and $\vec{q}$ is the molecular heatflux). Therefore, the analysis of entropy fluctuations is essential in understanding the thermodynamic turbulence structure of compressible aerodynamic flows.

Substantial progress has been made in recent years regarding the scaling of the meanflow in compressible aerodynamic wall turbulence [2,3,4] driven largely by the availability of detailed dns data [1,5,6,7,8,9,10,11]. However simulations at higher Reynolds numbers are needed to determine the high-Re asymptotics [12] of compressible wall turbulence [10]. These studies also demonstrate the success of hcb [13] inner scaling, based on the ★-system of units $\left\{{\bar{\tau }}_{w},\bar{\rho }\left(y\right),\bar{\mu }\left(y\right)\right\}$, where $\tau_{w}$ is the wall shear stress, *y* is the wall distance, ρ is the density and μ is the dynamic viscosity. We will denote {x,y,z} the Cartesian coordinates (streamwise, wall-normal and spanwise) with corresponding velocity components {u,v,w}, *p* the pressure, *T* the temperature and *s* the entropy, and adopt the notation $\left(\cdot \right)=\bar{\left(\cdot \right)}+{\left(\cdot \right)}^{\prime }$ for Reynolds averages and fluctuations. The subscript ${\left(\cdot \right)}_{w}$ denotes wall conditions, whereas the subscript ${\left(\cdot \right)}_{\mathrm{CL}}$ denotes, for plane channel flow, centreline conditions.

Mean velocity transformations [3] and hcb scaling [13] are quite successful in accounting for the density and temperature stratification [14] (pp. 119–138) (wall-normal *y*-gradients of $\bar{\rho }\left(y\right)$ and $\bar{T}\left(y\right)$ induced by the increase in the Mach number) on the mean and fluctuating velocity field, in line with Morkovin’s hypothesis [15]. However, regarding the fluctuating pressure $p^{\prime }$ [16], strong near-wall compressibility effects appear with increasing Mach number [17], but also with increasingly cold (relative to wall layer edge) wall temperature [18]. The combined effect of increasing Mach number and associated increase of centreline-to-wall temperature ratio ${\bar{T}}_{\mathrm{CL}}/{\bar{T}}_{w}$ induces increasing compressibility effects [19], essentially between the location of the out-of-the-wall $p_{\mathrm{rms}}^{\prime }$ peak and the wall, the outer part of the flow being less sensitive to the effects of compressibility.

The Z=1 equation-of-state (EoS) adopted in all of the above-cited studies [20] ((2.4), p. 452),

(1a) $$\begin{array}{ccc} p=\rho R_{g}T\Rightarrow \left(1+c_{\rho^{\prime }T^{\prime }}\;{\mathrm{CV}}_{\rho^{\prime }}\;{\mathrm{CV}}_{T^{\prime }}\right)\frac{p^{\prime }}{\bar{p}} & = & \frac{\rho^{\prime }}{\bar{\rho }}+\frac{T^{\prime }}{\bar{T}}+\frac{\rho^{\prime }T^{\prime }}{\bar{\rho }\bar{T}}-c_{\rho^{\prime }T^{\prime }}\;{\mathrm{CV}}_{\rho^{\prime }}\;{\mathrm{CV}}_{T^{\prime }} \end{array}$$

couples the turbulent fluctuations of the basic thermodynamic variables $\left\{p^{\prime },\rho^{\prime },T^{\prime }\right\}$. In (1a),

(1b) $$\begin{array}{c} {\mathrm{CV}}_{{\left(\cdot \right)}^{\prime }}:=\frac{{\left(\cdot \right)}_{\mathrm{rms}}^{\prime }}{\bar{\left(\cdot \right)}}\;;\;c_{{\left(\cdot \right)}^{\prime }{\left[\cdot \right]}^{\prime }}:=\frac{\bar{{\left(\cdot \right)}^{\prime }{\left[\cdot \right]}^{\prime }}}{\sqrt{\bar{{\left(\cdot \right)}^{\prime 2}}}\sqrt{\bar{{\left[\cdot \right]}^{\prime 2}}}}\;\in \left[-1,1\right]\;;\;{\left(\cdot \right)}_{\mathrm{rms}}^{\prime }:=\sqrt{\bar{{\left(\cdot \right)}^{\prime 2}}} \end{array}$$

denote the coefficients of variation (${\mathrm{CV}}_{p^{\prime }}$, ${\mathrm{CV}}_{\rho^{\prime }}$, ${\mathrm{CV}}_{T^{\prime }}$) and the correlation coefficient ($c_{\rho^{\prime }T^{\prime }}$). These relations (1) are the starting point to gain insight into the couplings between the fluctuations of thermodynamic variables via the EoS [20,21]. Often, the exact relation (1a) is linearised to develop simpler working approximations. In previous work, we systematically evaluated such approximate linearised relations between CVs and correlation coefficients (CCs) against dns data [20] and found that some of the approximations are particularly robust (remain accurate with increasing Mach number), including the approximation [20] ((4.5a), p. 461) for the fluctuating entropy $s_{\mathrm{rms}}^{\prime }$ in terms of (${\mathrm{CV}}_{p^{\prime }}$, ${\mathrm{CV}}_{\rho^{\prime }}$, ${\mathrm{CV}}_{T^{\prime }}$). On the contrary, nonlinear effects (increasing ${\mathrm{CV}}_{\rho^{\prime }}$ and/or ${\mathrm{CV}}_{T^{\prime }}$) are important in the approximations of correlations containing $p^{\prime }$ ($c_{s^{\prime }p^{\prime }}$,$c_{p^{\prime }\rho^{\prime }}$,$c_{p^{\prime }T^{\prime }}$) [20].

The entropy mode is one of the fundamental organised compressible turbulence mechanisms [22,23], and although entropy could be considered as a function of any pair of the basic thermodynamic variables, the statistical properties of $s^{\prime }$ are quite specific [20]. Analysis [20] of dns data [21] for sustained (large-scale solenoidal forcing) compressible homogeneous isotropic turbulence (hit) indicates that the entropy/temperature correlation coefficient is approximately constant, $c_{s^{\prime }T^{\prime }}\;\;≊\;\;0.2$. Data [20] for compressible turbulent plane channel (tpc) flow indicate that almost everywhere in the channel $y^{★}\;\;⪆\;\;10$, the entropy/pressure correlation is very weak, $|c_{s^{\prime }p^{\prime }}|\;\;⪅\;\;0.15$ [20] (Figure 14, p. 469). Both these observations lead to simple approximations for the thermodynamic turbulence structure in these flows [20]. Entropy fluctuations $s_{\mathrm{rms}}^{\prime }/R_{g}$ and correlations are central in these developments.

In the paper, we focus on compressible turbulent plane channel flow using dns data [10,11], which include detailed $s^{\prime }$ statistics. As noted earlier, HCB-scaling [13] is adopted for the Reynolds number and the wall distance. Regarding the Mach number, many authors use the bulk Mach number $M_{B_{w}}$ [5,13], but the wall layer edge Mach number is physically more relevant. We will therefore denote the turbulent plane channel flow conditions by the pair $\left(Re_{\tau^{★}},{\bar{M}}_{{\mathrm{CL}}_{x}}\right)$ of the hcb friction Reynolds number and streamwise centreline Mach number

(2) $$\begin{array}{c} y^{★}:=\frac{\bar{\rho }\left(y\right)}{\bar{\mu }\left(y\right)}\sqrt{\frac{{\bar{\tau }}_{w}}{\bar{\rho }\left(y\right)}}y\;;\;Re_{\tau^{★}}:=\delta^{★}=\frac{{\bar{\rho }}_{\mathrm{CL}}}{{\bar{\mu }}_{\mathrm{CL}}}\sqrt{\frac{{\bar{\tau }}_{w}}{{\bar{\rho }}_{\mathrm{CL}}}}\delta \;;\;{\bar{M}}_{{\mathrm{CL}}_{x}}:=\bar{\left(\frac{u_{\mathrm{CL}}}{a_{\mathrm{CL}}}\right)} \end{array}$$

and use $y^{★}$ as the inner-scaled non-dimensional wall-distance. Notice that in compressible turbulent plane channel flow, the relation between the bulk Mach number $M_{B_{w}}$ and the centreline streamwise Mach number ${\bar{M}}_{{\mathrm{CL}}_{x}}$ is strongly nonlinear, as it is depends on the intense frictional heating of the flow [11].

In Section 2 we briefly describe the dns data used in the present work. In Section 3, we concentrate on the rms-levels of $s^{\prime }$ across the channel, and examine in particular the dependence of the peak value ${\left[s_{\mathrm{rms}}^{\prime }\right]}_{\mathrm{PEAK}}$ on $\left(Re_{\tau^{★}},{\bar{M}}_{{\mathrm{CL}}_{x}}\right)$. In Section 4, we examine correlation coefficients ($c_{s^{\prime }p^{\prime }}$,$c_{s^{\prime }\rho^{\prime }}$,$c_{s^{\prime }T^{\prime }}$) which are analysed by examining the joint pdfs ($f_{p^{\prime }s^{\prime }},f_{\rho^{\prime }s^{\prime }},f_{T^{\prime }s}$). Higher-order statistics and probability density functions for $s^{\prime }$ are studied in Section 5. Conclusions and perspectives of the present work are summarised in Section 6. Finally, in the Appendix A, we work out the complete expansions of $s^{\prime }$ into power series of $\left(p^{\prime },T^{\prime }\right)$ or $\left(\rho^{\prime },T^{\prime }\right)$ for variable $c_{p}\left(T\right)$ thermodynamics, extending the relations in [20], and for future reference.

## 2. DNS Database

The dns database used in the present work was constructed by the authors [10,11] and is available at https://data.mendeley.com/datasets/wt8t5kxzbs/1 (accessed on 26 April 2024). It contains 25 $\left(Re_{\tau^{★}},{\bar{M}}_{{\mathrm{CL}}_{x}}\right)$ flow conditions covering $100\;\;⪅\;\;Re_{\tau^{★}}\;\;⪅\;\;1000$ and $0.3\;\;⪅\;\;{\bar{M}}_{{\mathrm{CL}}_{x}}\;\;⪅\;\;2.5$ and allowing for the examination of the ${\bar{M}}_{{\mathrm{CL}}_{x}}$-effect at nearly constant $Re_{\tau^{★}}$ and conversely.

The flow is modelled by the compressible Navier–Stokes equations using EoS (1a) with constant specific heat $c_{p}\;=\;\gamma R_{g}/\left(\gamma -1\right)\;\;=\;\;\mathrm{const}$. Details on the temperature dependence of viscosity μ(T) and heat conductivity λ(T) are given in [1] (p. 706) and bulk viscosity $\mu_{b}\;\;=\;\;0$.

The flow configuration is the canonical tpc flow configuration introduced by Coleman et al. [5], and was simulated using the dns solver described in [24]. Isothermal wall conditions are applied. Because of frictional heating, this is a very-cold-wall flow [11], and the centreline-to-wall temperature ratio ${\bar{T}}_{\mathrm{CL}}/{\bar{T}}_{w}$ is not a free parameter. The thermal wall condition is best characterised [11] by the non-dimensional wall-to-centreline enthalpy difference

(3) $$\begin{array}{c} r_{\bar{h}}:=\frac{{\bar{h}}_{w}-{\bar{h}}_{\mathrm{CL}}}{\frac{1}{2}{\bar{u}}_{\mathrm{CL}}^{2}} \end{array}$$

The present very-cold-wall data correspond to $0.58\;\;\leq \;\;-r_{\bar{h}}\;\;\leq \;\;0.65$. For flows with different $r_{\bar{h}}$, the turbulence structure of temperature fluctuations is modified [2], with substantial variations of the correlation coefficients $c_{T^{\prime }u^{\prime }}$ and $c_{T^{\prime }v^{\prime }}$, which change sign for hotter wall conditions [25]. The influence of wall temperature conditions on the thermodynamic turbulence structure requires specific study, either by using an artificial sink term in the energy equation for tpc flow [19], or by studying turbulent boundary layer (tbl) flow [8].

High-order accurate dns computations [24] were run for a sufficiently long physical time, eliminating the transient. Computations were continued acquiring moments for the computation of the turbulent correlations at each wall-normal station. Simultaneously extreme events were recorded at each wall-normal station. The values of these extreme events were used to determine the range of pdf bins, whose sampling started after an initial observation time (sufficiently long for a reasonable estimate of extreme events).

## 3. $s_{\mathrm{rms}}^{\prime }$

**Figure 1:**
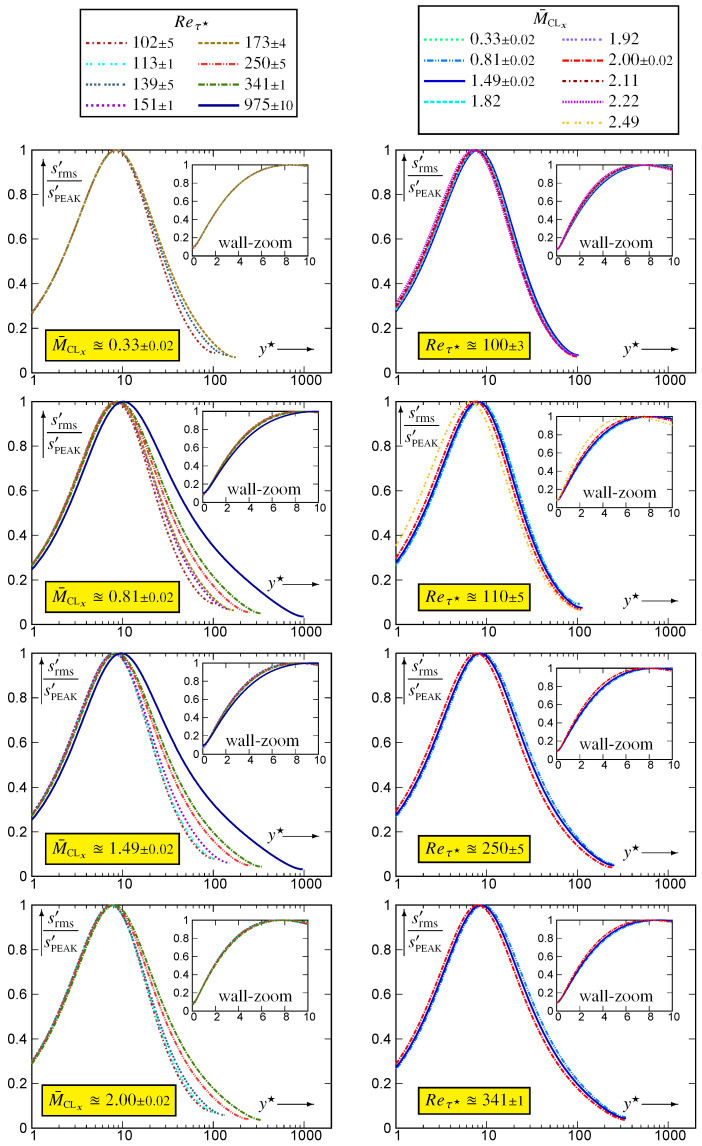
Profiles of entropy fluctuation rms scaled by its peak value, $s_{\mathrm{rms}}^{\prime }/{\left[s_{\mathrm{rms}}^{\prime }\right]}_{\mathrm{PEAK}}$, plotted against the HCB-scaled nondimensional wall distance $y^{★}$ (logscale), for varying $97\;\leq \;Re_{\tau^{★}}\;\leq \;983$ at nearly constant ${\bar{M}}_{{\mathrm{CL}}_{x}}\;\in \;\left\{0.33,0.80,1.50,2.00\right\}$, and for varying $0.32\;\leq \;{\bar{M}}_{{\mathrm{CL}}_{x}}\;\leq \;2.49$ at nearly constant $Re_{\tau^{★}}\;\in \;\left\{100,110,250,340\right\}$, using dns data [10,11].

The profiles of $s_{\mathrm{rms}}^{\prime }$ across the channel (Figure 1) reach a maximum, ${\left[s_{\mathrm{rms}}^{\prime }\right]}_{\mathrm{PEAK}}$ (4), very near the wall, then steadily drop to lower values towards the centreline. Let

(4) $$\begin{array}{c} {\left[s_{\mathrm{rms}}^{\prime }\right]}_{\mathrm{PEAK}}:=\max_{y}{s^{\prime }}_{\mathrm{rms}}\;;\;y_{s_{\mathrm{PEAK}}^{\prime }}^{★}:=y^{★}{|}_{{\left[s_{\mathrm{rms}}^{\prime }\right]}_{\mathrm{PEAK}}} \end{array}$$

Interpolation of the discrete data (degree-4 polynomial) in the neighbourhood of the discrete maximum was used to determine the location $y_{s_{\mathrm{PEAK}}^{\prime }}^{★}$ and value ${\left[s_{\mathrm{rms}}^{\prime }\right]}_{\mathrm{PEAK}}$ of the peak (4). Examination (Figure 1) of the $Re_{\tau^{★}}$-effect at nearly constant ${\bar{M}}_{{\mathrm{CL}}_{x}}$ indicates a displacement of the peak location away from the wall. This is especially visible for the $Re_{\tau^{★}}\;\;≊\;\;1000$ flows (${\bar{M}}_{{\mathrm{CL}}_{x}}\;\in \;\left\{0.81,1.50\right\}$) compared to $Re_{\tau^{★}}\;\;≊\;\;340$. There is no indication of near-wall behaviour since the profiles plotted against $y^{★}$ do not collapse on a single curve for $y^{★}<y_{s_{\mathrm{PEAK}}^{\prime }}^{★}$. Similar observations apply (Figure 1) to the behaviour of the profiles with varying ${\bar{M}}_{{\mathrm{CL}}_{x}}$ at nearly constant $Re_{\tau^{★}}$. The analysis of the $p^{\prime }$ behaviour at $\left(Re_{\tau^{★}},{\bar{M}}_{{\mathrm{CL}}_{x}}\right)\;=\;\left(113,2.49\right)$ [10,17] has revealed strong compressibility effects very close to the wall, and a strong ${\bar{M}}_{{\mathrm{CL}}_{x}}$-effect is also observed for $s_{\mathrm{rms}}^{\prime }$ ($Re_{\tau^{★}}≊110$; Figure 1).

**Figure 2:**
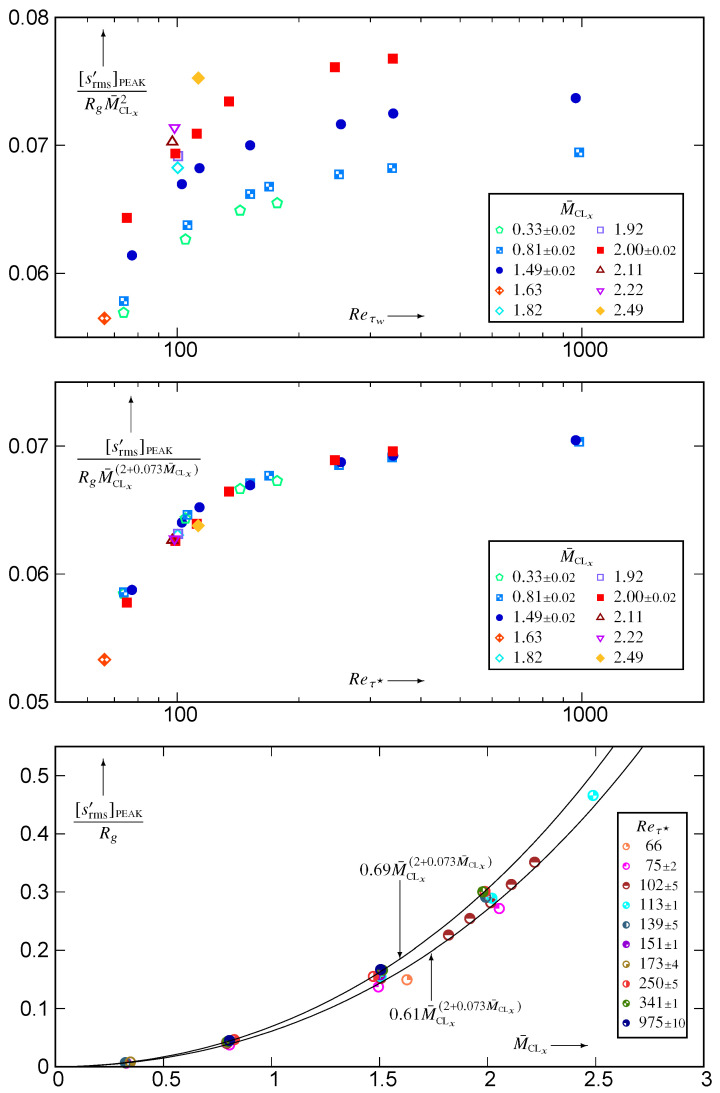
Peak level of nondimensional entropy fluctuation rms ${\left[s_{\mathrm{rms}}^{\prime }\right]}_{\mathrm{PEAK}}/R_{g}$, plotted against the hcb friction Reynolds number $Re_{\tau^{★}}$ for varying centreline Mach numbers $0.32\;\leq \;{\bar{M}}_{{\mathrm{CL}}_{x}}\;\leq \;2.49$ (using basic ${\bar{M}}_{{\mathrm{CL}}_{x}}^{2}$ or improved ${\bar{M}}_{{\mathrm{CL}}_{x}}^{\left(2+0.073{\bar{M}}_{{\mathrm{CL}}_{x}}\right)}$ Mach-scalings), and against ${\bar{M}}_{{\mathrm{CL}}_{x}}$ for varying $66\;\leq \;Re_{\tau^{★}}\;\leq \;983$ (with envelop of ${\bar{M}}_{{\mathrm{CL}}_{x}}$-scaling), using dns data [10,11].

The expansions of $s^{\prime }$ (A6c, A9c) highlight the fact that $s_{\mathrm{rms}}^{\prime }/R_{g}$ is nondimensional. The limited number of low-$Re_{\tau^{★}}$ flows examined in [1] suggest that $s_{\mathrm{rms}}^{\prime }/R_{g}$ scales roughly but not exactly as ${\bar{M}}_{{\mathrm{CL}}_{x}}^{2}$. Plotting ${\left[s_{\mathrm{rms}}^{\prime }\right]}_{\mathrm{PEAK}}/\left(R_{g}{\bar{M}}_{{\mathrm{CL}}_{x}}^{2}\right)$ *vs* $Re_{\tau^{★}}$ (Figure 2) for all available data [10,11] shows that although ${\bar{M}}_{{\mathrm{CL}}_{x}}^{2}$-scaling brings closer together the data for different ${\bar{M}}_{{\mathrm{CL}}_{x}}$, there still remains a distinct ${\bar{M}}_{{\mathrm{CL}}_{x}}$-effect, and indeed, a polynomial ${\bar{M}}_{{\mathrm{CL}}_{x}}$-scaling of ${\left[s_{\mathrm{rms}}^{\prime }\right]}_{\mathrm{PEAK}}/R_{g}$ does not seem to fit the data. Using an ${\bar{M}}_{{\mathrm{CL}}_{x}}$-dependent exponent of ${\bar{M}}_{{\mathrm{CL}}_{x}}$ very satisfactorily collapses all data for ${\left[s_{\mathrm{rms}}^{\prime }\right]}_{\mathrm{PEAK}}/\left(R_{g}{\bar{M}}_{{\mathrm{CL}}_{x}}^{\left(2+0.073{\bar{M}}_{{\mathrm{CL}}_{x}}\right)}\right)$ on a single $Re_{\tau^{★}}$-dependent curve (Figure 2). Notice that a similar ${\bar{M}}_{{\mathrm{CL}}_{x}}$-dependent exponent of ${\bar{M}}_{{\mathrm{CL}}_{x}}$ was found necessary to fit the ratio of adiabatic recovery temperature $T_{r}/{\bar{T}}_{w}$ in [10] (Figure 6, p. A19-15). This ${\bar{M}}_{{\mathrm{CL}}_{x}}$-dependence is further illustrated by plotting ${\left[s_{\mathrm{rms}}^{\prime }\right]}_{\mathrm{PEAK}}$ *vs* ${\bar{M}}_{{\mathrm{CL}}_{x}}$ (Figure 2), along with the envelope corresponding to the low ($Re_{\tau^{★}}≊100$) and high ($Re_{\tau^{★}}≊1000$) Reynolds numbers.

**Figure 3:**
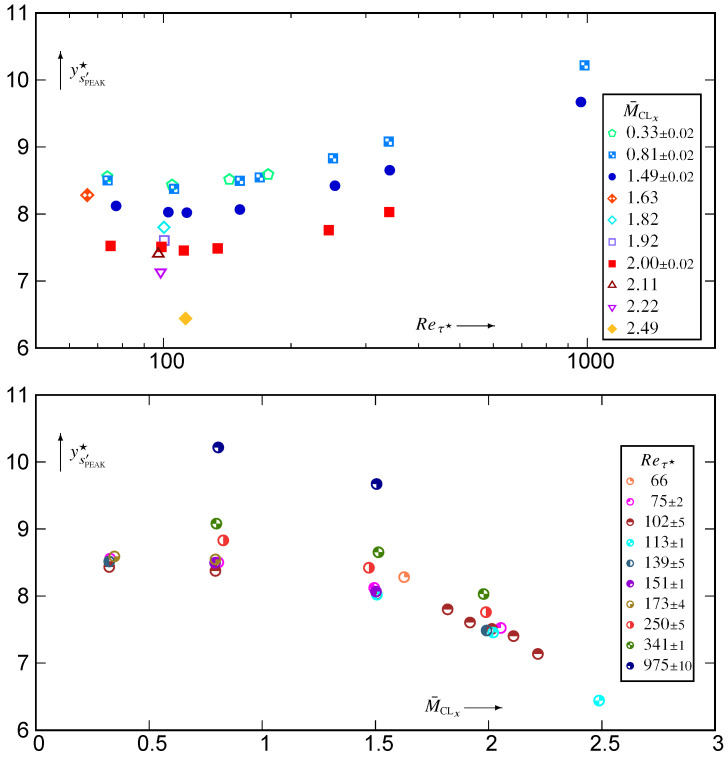
HCB-scaled nondimensional distance from the wall of the $s_{\mathrm{rms}}^{\prime }$ peak location, $y_{s_{\mathrm{PEAK}}^{\prime }}^{★}$, plotted against the hcb friction Reynolds number $Re_{\tau^{★}}$ for varying centreline Mach numbers $0.32\;\leq \;{\bar{M}}_{{\mathrm{CL}}_{x}}\;\leq \;2.49$, and against ${\bar{M}}_{{\mathrm{CL}}_{x}}$ for varying $66\;\leq \;Re_{\tau^{★}}\;\leq \;983$, using dns data [10,11].

Examination of the location $y_{s_{\mathrm{PEAK}}^{\prime }}^{★}$ plotted against $Re_{\tau^{★}}$ (Figure 3) quantifies the moving away from the wall of the ${\left[s_{\mathrm{rms}}^{\prime }\right]}_{\mathrm{PEAK}}$ for $Re_{\tau^{★}}\;>\;100$, the subcritical transitional flows ($Re_{\tau^{★}}\;<\;100$) having the opposite behaviour. Regarding the influence of ${\bar{M}}_{{\mathrm{CL}}_{x}}$, $y_{s_{\mathrm{PEAK}}^{\prime }}^{★}$ diminishes noticeably with increasing supersonic ${\bar{M}}_{{\mathrm{CL}}_{x}}\;>\;1$. This contrasts the behaviour of $y_{p_{\mathrm{PEAK}}^{\prime }}^{★}$, which moves away from the wall with increasing ${\bar{M}}_{{\mathrm{CL}}_{x}}$ [10] (Figure 5, p. A19-14).

## 4. Correlation of $s^{\prime }$ with $\left\{p^{\prime },\rho^{\prime },T^{\prime }\right\}$

Entropy can be computed from its definition as a state variable, Tds=de+pd(1/ρ)[26] (pp. 1–38), where *e* is the internal energy. Combined with the EoS (1a), this relation can be used to express $s^{\prime }/R_{g}$ as infinite power-series of $\rho^{\prime }$ and $T^{\prime }$, and this was performed in the Appendix A (A9c) for the general case of variable $c_{p}\left(T\right)$.

The leading terms of this expression have been used in [20] ((4.5a), p. 461) and combined to the expression of $p^{\prime }$ (1a) to show that, to leading order, the nondimensional

(5) $$\begin{array}{c} \frac{s_{\mathrm{rms}}^{\prime }}{R_{g}}∼\sqrt{\frac{\gamma }{{\left(\gamma -1\right)}^{2}}\;{\mathrm{CV}}_{T^{\prime }}^{2}+\frac{\gamma }{\gamma -1}\;{\mathrm{CV}}_{\rho^{\prime }}^{2}-\frac{1}{\gamma -1}\;{\mathrm{CV}}_{p^{\prime }}^{2}+\mathrm{HoTs}} \end{array}$$

is the square root of a weighted combination of (${\mathrm{CV}}_{p^{\prime }},{\mathrm{CV}}_{\rho^{\prime }},{\mathrm{CV}}_{T^{\prime }})$, with HoTs denoting higher-order terms (higher powers of (${\mathrm{CV}}_{p^{\prime }},{\mathrm{CV}}_{\rho^{\prime }},{\mathrm{CV}}_{T^{\prime }})$). Relation (5) was compared with dns data in [20], and is quite accurate, even for the higher ${\bar{M}}_{{\mathrm{CL}}_{x}}\;\;=\;\;2.49$ [20] (Figure 8, p. 465).

Most dns computations use a strictly isothermal wall condition, and even adiabatic-wall turbulent boundary layer simulations usually apply an isothermal wall condition at the theoretical adiabatic-wall recovery temperature $T_{r}$. This condition implies

(6a) $$\begin{array}{cc} & \mathrm{strictly}\;\mathrm{isothermal}\;\mathrm{wall}:\\ & T_{w}=\mathrm{const}\;\forall \;t\Rightarrow T_{w}^{\prime }=0\;\forall \;t\overset{\left(1a\right)}{\Rightarrow }{\left(\frac{p^{\prime }}{\bar{p}}\right)}_{w}\;\;=\;\;{\left(\frac{\rho^{\prime }}{\bar{\rho }}\right)}_{w}\;\;\Rightarrow \;\;{\left[{\mathrm{CV}}_{p^{\prime }}\right]}_{w}={\left[{\mathrm{CV}}_{\rho^{\prime }}\right]}_{w}\;\overset{\left(5\right)}{≊}\;{\left(\frac{s_{\mathrm{rms}}^{\prime }}{R_{g}}\right)}_{w} \end{array}$$

**Figure 4:**
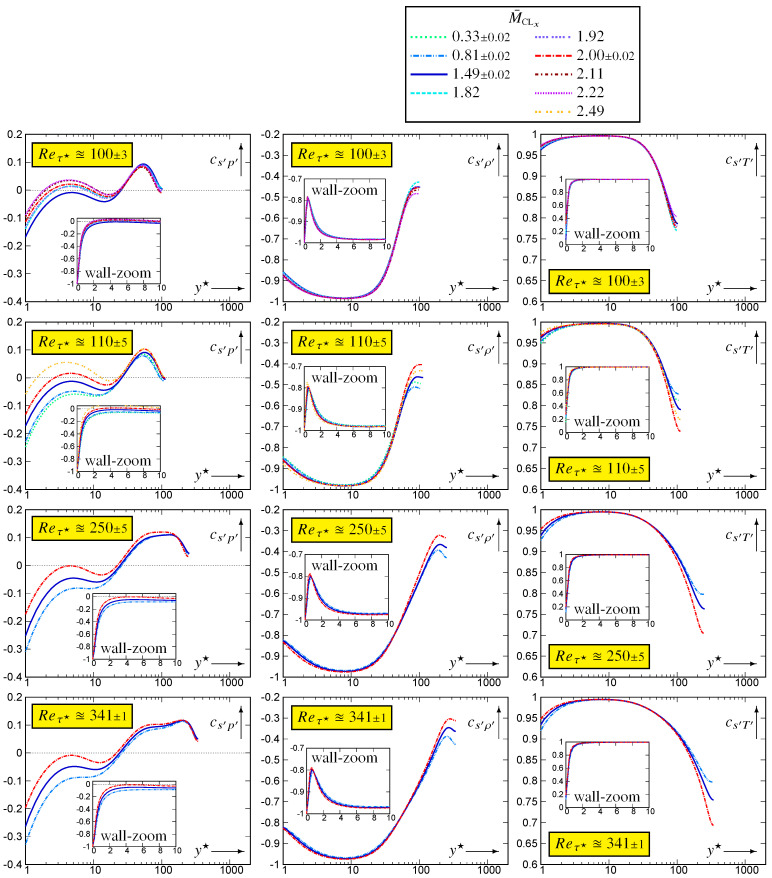
Correlation coefficients $\left\{c_{s^{\prime }p^{\prime }},c_{s^{\prime }\rho^{\prime }},c_{s^{\prime }T^{\prime }}\right\}$ of entropy fluctuations with the fluctuations of the basic thermodynamic quantities, plotted against the HCB-scaled nondimensional wall distance $y^{★}$ (logscale), for varying $0.32\;\;\leq \;\;{\bar{M}}_{{\mathrm{CL}}_{x}}\;\;\leq \;\;2.49$ at nearly constant $Re_{\tau^{★}}\;\in \;\left\{100,110,250,340\right\}$, using dns data [10,11].

**Figure 5:**
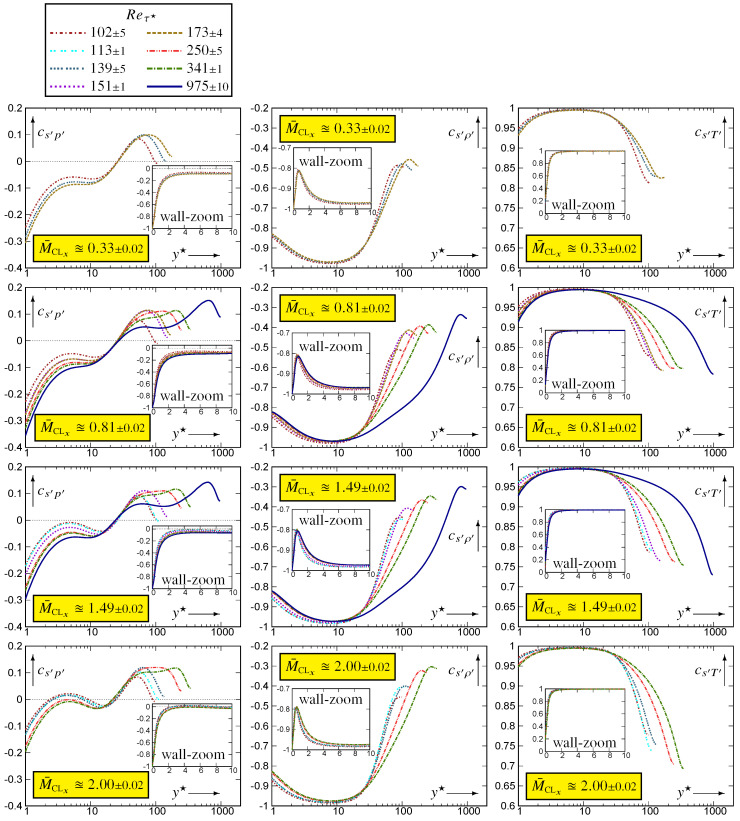
Correlation coefficients $\left\{c_{s^{\prime }p^{\prime }},c_{s^{\prime }\rho^{\prime }},c_{s^{\prime }T^{\prime }}\right\}$ of entropy fluctuations with the fluctuations of the basic thermodynamic quantities, plotted against the HCB-scaled nondimensional wall distance $y^{★}$ (logscale), for $97\;\;\leq \;\;Re_{\tau^{★}}\;\;\leq \;\;985$ at nearly constant ${\bar{M}}_{{\mathrm{CL}}_{x}}\;\in \;\left\{0.33,0.80,1.50,2.00\right\}$, using dns data [10,11].

The wall zoom of correlations $\left\{c_{s^{\prime }p^{\prime }},c_{s^{\prime }\rho^{\prime }},c_{s^{\prime }T^{\prime }}\right\}$ Figure 4 and Figure 5 clearly shows the effect of the strictly isothermal-wall condition

(6b) $$\begin{array}{c} \left(6a,A6c,A9c\right)\Rightarrow \left\{\begin{array}{c} {\left[c_{s^{\prime }T^{\prime }}\right]}_{w}=0\\ {\left[c_{s^{\prime }p^{\prime }}\right]}_{w}={\left[c_{s^{\prime }\rho^{\prime }}\right]}_{w}=-1 \end{array}\right. \end{array}$$

which is confined very near the wall ($y^{★}\;<\;2$).

Further away from the wall $-0.15\;\;⪅\;\;c_{s^{\prime }p^{\prime }}\;\;⪅\;\;0.15$, in line with similar observations in [20], which led to the quite successful $c_{s^{\prime }p^{\prime }}\;\;≊\;\;0$ approximation for the thermodynamic turbulence structure. Notice, nonetheless, that the higher $Re_{\tau^{★}}\;\;≊\;\;1000$ data (Figure 5) show a very slight increasing trend at the beginning of the wake region, which roughly corresponds to $\max_{y}c_{s^{\prime }p^{\prime }}$. Notice also the expected [10] very significant ${\bar{M}}_{{\mathrm{CL}}_{x}}$-effects on $c_{s^{\prime }p^{\prime }}$ in the near-wall zone ($2\;\;⪅\;\;y^{★}\;\;⪅\;\;20$; Figure 4).

In contrast to $c_{s^{\prime }p^{\prime }}$, both $c_{s^{\prime }\rho^{\prime }}$ and $c_{s^{\prime }T^{\prime }}$ exhibit a large near-wall zone ($5\;\;⪅\;\;y^{★}\;\;⪅\;\;100$) where there is very small ${\bar{M}}_{{\mathrm{CL}}_{x}}$-effect at nearly constant $Re_{\tau^{★}}$ (Figure 4). There is, however, noticeable ${\bar{M}}_{{\mathrm{CL}}_{x}}$-variation in the wake region for both $c_{s^{\prime }\rho^{\prime }}$ and $c_{s^{\prime }T^{\prime }}$ (Figure 4). With increasing $Re_{\tau^{★}}$ at nearly constant ${\bar{M}}_{{\mathrm{CL}}_{x}}$, the region of strong positive $c_{s^{\prime }T^{\prime }}\;\;⪆\;\;0.95$ correlation increases, probably until the beginning of the log-region (Figure 5).

The behaviour of $c_{s^{\prime }\rho^{\prime }}$ and $c_{s^{\prime }T^{\prime }}$ is better understood using the EoS (1a). Multiplying (1a) by $s^{\prime }$ and averaging yields the exact relation

(7a) $$\begin{array}{cc} c_{s^{\prime }p^{\prime }}\;{\mathrm{CV}}_{p^{\prime }}\overset{\left(1a\right)}{=} & c_{s^{\prime }\rho^{\prime }}\;{\mathrm{CV}}_{\rho^{\prime }}+c_{s^{\prime }T^{\prime }}\;{\mathrm{CV}}_{T^{\prime }}+\overset{\mathrm{HoTs}}{\overset{︷}{c_{s^{\prime }\rho^{\prime }T^{\prime }}{\mathrm{CV}}_{\rho^{\prime }}{\mathrm{CV}}_{T^{\prime }}-c_{s^{\prime }p^{\prime }}{\mathrm{CV}}_{p^{\prime }}{\mathrm{CV}}_{\rho^{\prime }}{\mathrm{CV}}_{T^{\prime }}}} \end{array}$$

where the correlation coefficient $c_{s^{\prime }\rho^{\prime }T^{\prime }}\;:=\;\bar{s^{\prime }\rho^{\prime }T^{\prime }}/\left(s_{\mathrm{rms}}^{\prime }\rho_{\mathrm{rms}}^{\prime }T_{\mathrm{rms}}^{\prime }\right)$, and to leading order [20] ((4.6c), p. 461)

(7b) $$\begin{array}{cc} c_{s^{\prime }p^{\prime }}\;{\mathrm{CV}}_{p^{\prime }}\overset{\left(7a\right)}{∼} & c_{s^{\prime }\rho^{\prime }}\;{\mathrm{CV}}_{\rho^{\prime }}+c_{s^{\prime }T^{\prime }}\;{\mathrm{CV}}_{T^{\prime }}+O\left({\mathrm{CV}}_{\rho^{\prime }}\;{\mathrm{CV}}_{T^{\prime }}\right) \end{array}$$

The error in the leading-order relation (7b) contains products of CVs (not rational combinations), and therefore, approximation (7b) is expected to be robust. By (7b), in a large part of the channel where $|c_{s^{\prime }p^{\prime }}{\mathrm{CV}}_{p^{\prime }}/{\mathrm{CV}}_{\rho^{\prime }}|\ll 1$, the ratio $c_{s^{\prime }\rho^{\prime }}/c_{s^{\prime }T^{\prime }}\;≊-{\mathrm{CV}}_{T}/{\mathrm{CV}}_{\rho^{\prime }}\;<\;0$.

**Figure 6:**
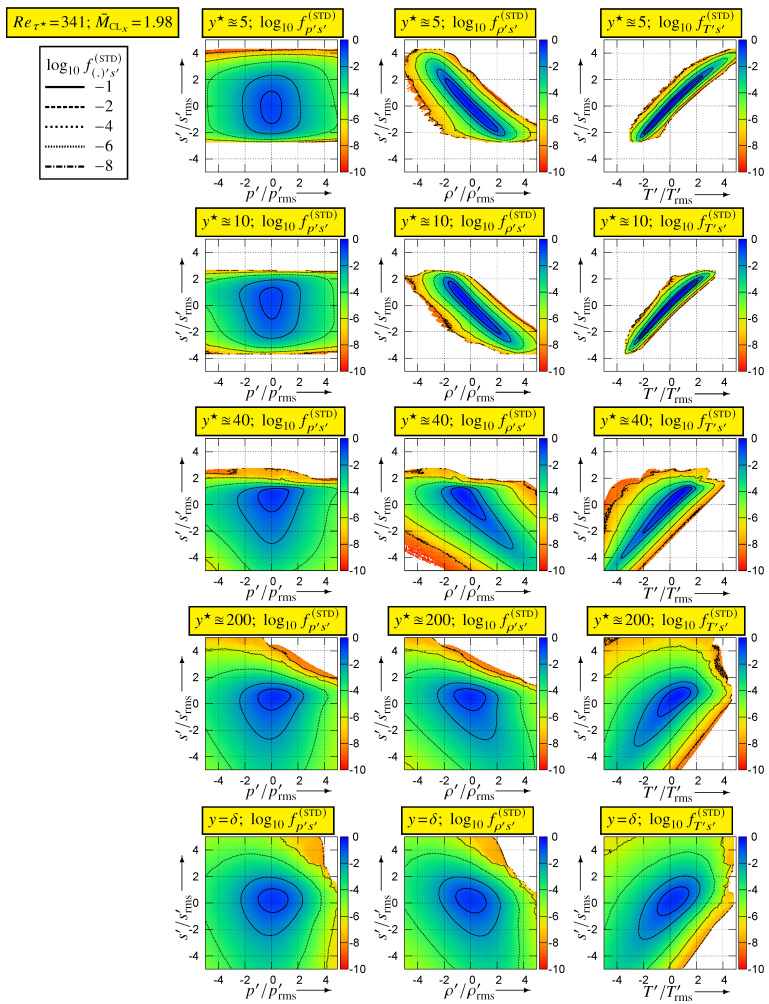
Logarithm of standardised joint pdfs of entropy $s^{\prime }$ and the basic thermodynamic variables ($\log_{10}f_{p^{\prime }s^{\prime }}^{\left(\mathrm{STD}\right)}$, $\log_{10}f_{\rho^{\prime }s^{\prime }}^{\left(\mathrm{STD}\right)}$, $\log_{10}f_{T^{\prime }s^{\prime }}^{\left(\mathrm{STD}\right)}$), plotted against the standardised variables ($s^{\prime }/s_{\mathrm{rms}}^{\prime }$, $p^{\prime }/p_{\mathrm{rms}}^{\prime }$, $\rho^{\prime }/\rho_{\mathrm{rms}}^{\prime }$, $T^{\prime }/T_{\mathrm{rms}}^{\prime }$), for different inner-scaled wall distances $y^{★}\;\in \;\left\{5.09,9.77,41.42,199.02,340.99=\delta^{★}\right\}$, at $\left(Re_{\tau^{★}},{\bar{M}}_{{\mathrm{CL}}_{x}}\right)\;=\;\left(341,1.98\right)$, using dns data [10,11].

**Figure 7:**
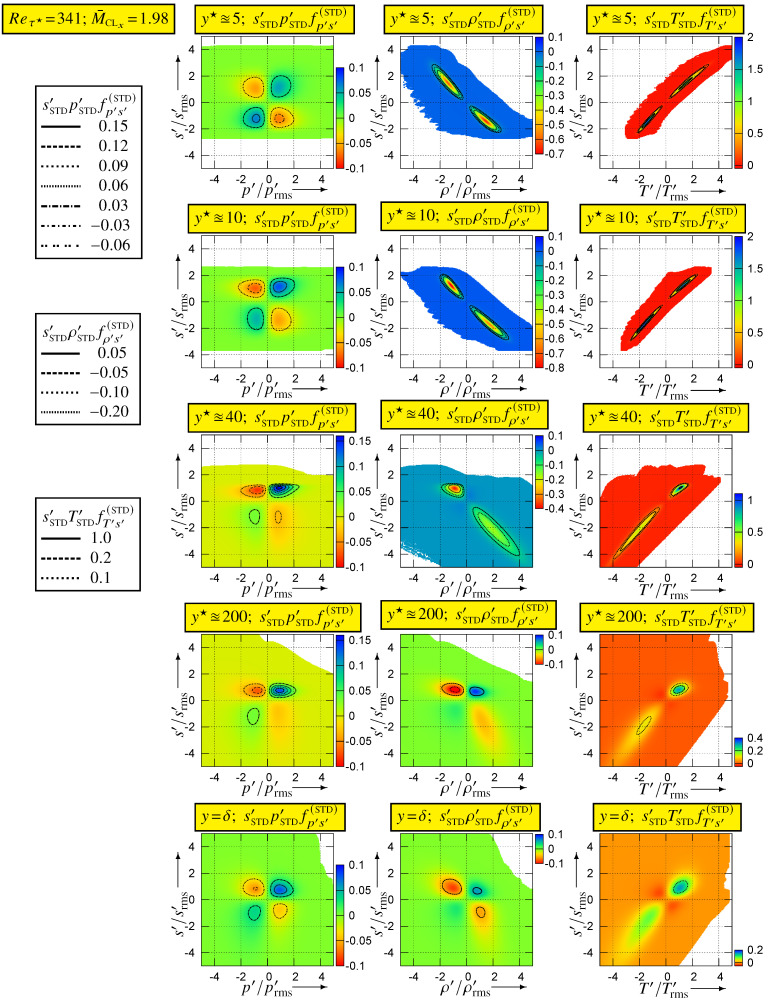
Integrands (8) for the calculation of the correlation coefficients $\left\{c_{s^{\prime }p^{\prime }},c_{s^{\prime }\rho^{\prime }},c_{s^{\prime }T^{\prime }}\right\}$ from the corresponding joint pdfs (Figure 6), plotted against the standardised variables ($s^{\prime }/s_{\mathrm{rms}}^{\prime }$, $p^{\prime }/p_{\mathrm{rms}}^{\prime }$, $\rho^{\prime }/\rho_{\mathrm{rms}}^{\prime }$, $T^{\prime }/T_{\mathrm{rms}}^{\prime }$), for different inner-scaled wall distances $y^{★}\;\in \;\left\{5.09,9.77,41.42,199.02,340.99=\delta^{★}\right\}$, at $\left(Re_{\tau^{★}},{\bar{M}}_{{\mathrm{CL}}_{x}}\right)\;=\;\left(341,1.98\right)$, using dns data [10,11].

Further insight into the correlation coefficients is obtained by studying the joint pdfs of $s^{\prime }$ with the other thermodynamic variables (Figure 6) and the integrands (Figure 7) for the evaluation of the correlation coefficients from the joint pdfs

(8a) $$\begin{array}{c} c_{s^{\prime }p^{\prime }}=\int_{s_{{\mathrm{STD}}_{\min}}^{\prime }}^{s_{{\mathrm{STD}}_{\max}}^{\prime }}\int_{p_{{\mathrm{STD}}_{\min}}^{\prime }}^{p_{{\mathrm{STD}}_{\max}}^{\prime }}s_{\mathrm{STD}}^{\prime }p_{\mathrm{STD}}^{\prime }\;f_{s^{\prime }p^{\prime }}^{\left(\mathrm{STD}\right)}\left(s_{\mathrm{STD}}^{\prime },p_{\mathrm{STD}}^{\prime }\right)\;ds_{\mathrm{STD}}^{\prime }\;dp_{\mathrm{STD}}^{\prime } \end{array}$$

(8b) $$\begin{array}{c} c_{s^{\prime }\rho^{\prime }}=\int_{s_{{\mathrm{STD}}_{\min}}^{\prime }}^{s_{{\mathrm{STD}}_{\max}}^{\prime }}\int_{\rho_{{\mathrm{STD}}_{\min}}^{\prime }}^{\rho_{{\mathrm{STD}}_{\max}}^{\prime }}s_{\mathrm{STD}}^{\prime }\rho_{\mathrm{STD}}^{\prime }\;f_{s^{\prime }\rho^{\prime }}^{\left(\mathrm{STD}\right)}\left(s_{\mathrm{STD}}^{\prime },\rho_{\mathrm{STD}}^{\prime }\right)\;ds_{\mathrm{STD}}^{\prime }\;d\rho_{\mathrm{STD}}^{\prime } \end{array}$$

(8c) $$\begin{array}{c} c_{s^{\prime }T^{\prime }}=\int_{s_{{\mathrm{STD}}_{\min}}^{\prime }}^{s_{{\mathrm{STD}}_{\max}}^{\prime }}\int_{T_{{\mathrm{STD}}_{\min}}^{\prime }}^{T_{{\mathrm{STD}}_{\max}}^{\prime }}s_{\mathrm{STD}}^{\prime }T_{\mathrm{STD}}^{\prime }\;f_{s^{\prime }T^{\prime }}^{\left(\mathrm{STD}\right)}\left(s_{\mathrm{STD}}^{\prime },T_{\mathrm{STD}}^{\prime }\right)\;ds_{\mathrm{STD}}^{\prime }\;dT_{\mathrm{STD}}^{\prime } \end{array}$$

integrated with respect to the standardised variables

(8d) $$\begin{array}{c} s_{\mathrm{STD}}^{\prime }:=s^{\prime }/s_{\mathrm{rms}}^{\prime }\;;\;p_{\mathrm{STD}}^{\prime }:=p^{\prime }/p_{\mathrm{rms}}^{\prime }\;;\;\rho_{\mathrm{STD}}^{\prime }:=\rho^{\prime }/\rho_{\mathrm{rms}}^{\prime }\;;\;T_{\mathrm{STD}}^{\prime }:=T^{\prime }/T_{\mathrm{rms}}^{\prime } \end{array}$$

For $\left(Re_{\tau^{★}},{\bar{M}}_{{\mathrm{CL}}_{x}}\right)\;=\;\left(341,1.98\right)$, $t_{{\mathrm{OBS}}_{2q}}\;=\;3854$ with sampling at every iteration, so that the joint pdfs were calculated at each $y^{★}$-station from $∼\;477\;\;\times \;\;10^{9}$ events (computational grid $N_{x}\times N_{y}\times N_{z}=1441\times 281\times 2001$ with upper/lower half-channel-averaging, i.e., 560,000 samples per time-step).

The joint pdfs (Figure 6) and the integrands (Figure 7) for $\left(Re_{\tau^{★}},{\bar{M}}_{{\mathrm{CL}}_{x}}\right)\;=\;\left(341,1.98\right)$ highlight the major difference between $c_{s^{\prime }p^{\prime }}$ and the two other correlations. The joint pdf $f_{s^{\prime }p^{\prime }}$ is quite symmetric around the origin, where it is approximately maximal (Figure 6), so that the $c_{s^{\prime }p^{\prime }}$-integrands (8a) are nearly symmetric with respect to the vertical ($s^{\prime }$) axis, with small value in each quadrant. Therefore, the integral in each $(p^{\prime }\;<\;0$)-quadrant nearly cancels the integral in the corresponding $\left(p^{\prime }\;>\;0\right)$-quadrant yielding small values for $|c_{s^{\prime }p^{\prime }}|$ (Figure 4). In contrast (Figure 6), both $f_{s^{\prime }\rho^{\prime }}$ and $f_{s^{\prime }T^{\prime }}$ have high values (recall that $\log_{10}f_{{\left(.\right)}^{\prime }{\left[.\right]}^{\prime }}^{\left(\mathrm{STD}\right)}$ is plotted) clustered along the diagonals of the negative ($f_{s^{\prime }\rho^{\prime }}$) and positive ($f_{s^{\prime }T^{\prime }}$) quadrants, respectively. Near the wall ($y^{★}\;\in \;\left\{5,10\right\}$), this clustering along the diagonal is very tight, especially for $T^{\prime }$ (Figure 6). Therefore, for $y^{★}\;\in \;\left\{5,10\right\}$, the $c_{s^{\prime }T^{\prime }}$-integrand takes quite high positive values, tightly clustered along the positive diagonal (Figure 7), resulting in $c_{s^{\prime }T^{\prime }}$ being very close to 1 at these $y^{★}$ (Figure 4). It is noticeable how negligibly small the $c_{s^{\prime }T^{\prime }}$-integrand is in the negative quadrant, for $y^{★}\;\in \;\left\{5,10\right\}$ (Figure 7).

The domain covered by the bins used for the sampling of the joint pdfs was square (limited in the range $\pm 5{\left[\cdot \right]}_{\mathrm{rms}}^{\prime }$ for each variable). When plotting $\log_{10}f_{s^{\prime }{\left[.\right]}^{\prime }}^{\left(\mathrm{STD}\right)}$ (Figure 6) or the integrands (Figure 7), bins for which no events were observed were left blank. Therefore, no events were observed outside of the coloured area (Figure 6 and Figure 7) during the joint pdfs sampling time $t_{{\mathrm{OBS}}_{2q}}$, the pdf $f_{s^{\prime }{\left[.\right]}^{\prime }}^{\left(\mathrm{STD}\right)}$ having generally dropped below $10^{-9}$ at the end of the coloured space. This implies that, for $y^{★}\in \left\{5,10\right\}$, $\left(T^{\prime },s^{\prime }\right)$-events only occur very close to the positive diagonal and that extreme $s^{\prime }$-events are not so extreme with respect to $s_{\mathrm{rms}}^{\prime }$ (Section 5). Moving further away from the wall, $\left(s^{\prime },T^{\prime }\right)$-events occur progressively further away from the diagonal ($y^{★}\;\in \;\left\{40,200,341\right\}$; Figure 6) so that the $c_{s^{\prime }T^{\prime }}$-integrands (Figure 7) take lower values maintaining nevertheless an overwhelming dominance of the positive $\left(s^{\prime }T^{\prime }\;>\;0\right)$-quadrants. As a result (Figure 4), $c_{s^{\prime }T^{\prime }}$ decreases with increasing $y^{★}$.

Exactly the same observations as for $f_{s^{\prime }T^{\prime }}$ apply for $f_{s^{\prime }\rho^{\prime }}$ (Figure 6) and for the $c_{s^{\prime }\rho^{\prime }}$-integrand (Figure 7), but this time, events are clustered along the diagonal of the negative quadrants (Figure 6) and the $c_{s^{\prime }\rho^{\prime }}$-integrand is dominated by the negative $\left(s^{\prime }\rho^{\prime }\;<\;0\right)$-quadrants (Figure 7). Notice, however, that even near the wall ($y^{★}\;\in \;\left\{5,10\right\}$), the clustering of $f_{s^{\prime }\rho^{\prime }}$ around the diagonal of the negative $\left(s^{\prime }\rho^{\prime }\;\;<\;0\right)$-quadrants is less tight than that of $f_{s^{\prime }T^{\prime }}$ around the diagonal of the positive $\left(s^{\prime }T^{\prime }\;>\;0\right)$-quadrants (Figure 6). This explains the very strong negative correlation $c_{s^{\prime }\rho^{\prime }}$ near the wall and the fact that this correlation is slightly weaker than $c_{s^{\prime }T^{\prime }}$ (Figure 4 and Figure 7). With increasing distance from the wall, positive contributions from the $\left(\rho^{\prime }\;>\;0,s^{\prime }\;>\;0\right)$-quadrant (Figure 7) reduce the anticorrelation $c_{s^{\prime }\rho^{\prime }}$ compared to $c_{s^{\prime }T^{\prime }}$ (7b). With increasing wall distance [20] (Figure 3, p. 458), ${\mathrm{CV}}_{T^{\prime }}/{\mathrm{CV}}_{\rho^{\prime }}$ decreases, and the correlation coefficient $c_{s^{\prime }p^{\prime }}$ is very weak in the outer part of the flow (Figure 5). Therefore, by (7b) $c_{s^{\prime }\rho^{\prime }}$ is close to $-\frac{1}{2}c_{s^{\prime }T^{\prime }}$ in the centreline region (Figure 5).

## 5. Higher-Order Statistics

**Figure 8:**
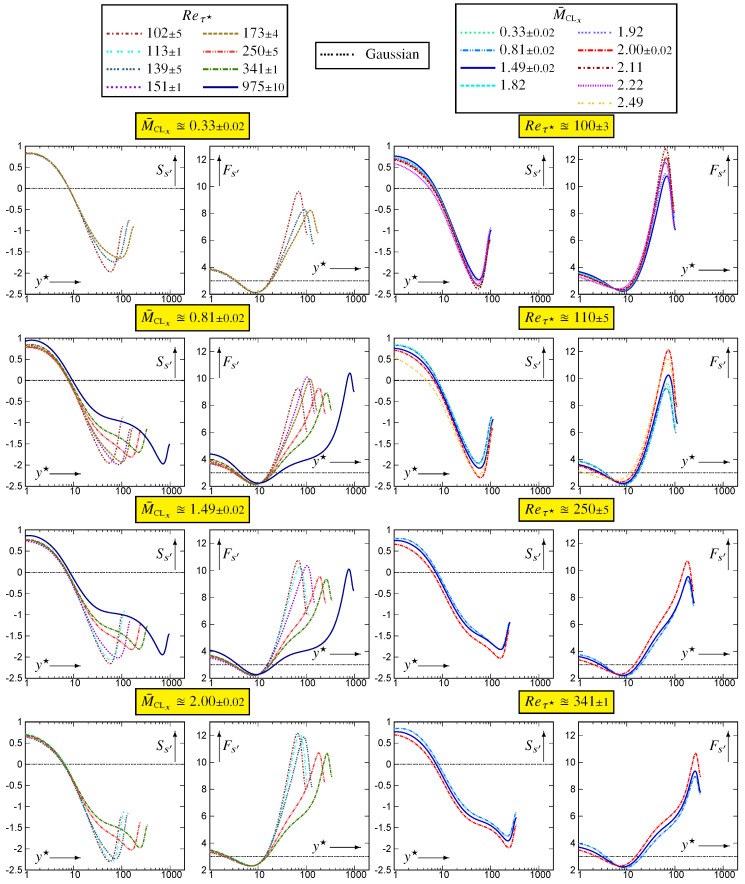
Profiles of skewness $S_{s^{\prime }}\;:=\;\bar{s^{\prime 3}}/s_{\mathrm{rms}}^{\prime 3}$ and flatness $F_{s^{\prime }}\;:=\;\bar{s^{\prime 4}}/s_{\mathrm{rms}}^{\prime 4}$ of entropy fluctuations, plotted against the HCB-scaled nondimensional wall-distance $y^{★}$ (logscale), for varying $97\;\;\leq \;\;Re_{\tau^{★}}\;\;\leq \;\;983$ at nearly constant ${\bar{M}}_{{\mathrm{CL}}_{x}}\;\in \;\left\{0.33,0.80,1.50,2.00\right\}$, and for varying $0.32\leq {\bar{M}}_{{\mathrm{CL}}_{x}}\;\;\leq \;\;2.49$ at nearly constant $Re_{\tau^{★}}\;\in \;\left\{100,110,250,340\right\}$, using dns data [10,11].

Although rms-levels and CCs (including joint pdfs) are important, the actual behaviour of the fluctuating field is better understood by extreme events and associated higher-order moments. Skewness $S_{s^{\prime }}$ (9b) and flatness $F_{s^{\prime }}$ (9c) depend on both ${\bar{M}}_{{\mathrm{CL}}_{x}}$ and $Re_{\tau^{★}}$ everywhere in the channel (Figure 8). They were calculated from the standardised pdf of entropy which was acquired at every wall-normal location of the computational grid.

(9a) $$\begin{array}{c} 1=\int_{s_{\min}^{\prime }}^{s_{\max}^{\prime }}f_{s^{\prime }}\left(s^{\prime }\right)\;ds^{\prime }=\int_{s_{\min}^{\prime }}^{s_{\max}^{\prime }}\overset{f_{s^{\prime }}^{\left(\mathrm{STD}\right)}}{\overset{︷}{s_{\mathrm{rms}}^{\prime }f_{s^{\prime }}}}\;d\overset{s_{\mathrm{STD}}^{\prime }}{\overset{︷}{\left(\frac{s^{\prime }}{s_{\mathrm{rms}}^{\prime }}\right)}}=\int_{s_{{\mathrm{STD}}_{\min}}^{\prime }}^{s_{{\mathrm{STD}}_{\max}}^{\prime }}f_{s^{\prime }}^{\left(\mathrm{STD}\right)}\;ds_{\mathrm{STD}}^{\prime } \end{array}$$

(9b) $$\begin{array}{c} S_{s^{\prime }}:=\frac{\bar{s^{\prime 3}}}{s_{\mathrm{rms}}^{\prime 3}}=\int_{s_{\min}^{\prime }}^{s_{\max}^{\prime }}\frac{s^{\prime 3}\;f_{s^{\prime }}}{s_{\mathrm{rms}}^{\prime 2}}\;d\left(\frac{s^{\prime }}{s_{\mathrm{rms}}^{\prime }}\right)=\int_{s_{{\mathrm{STD}}_{\min}}^{\prime }}^{s_{{\mathrm{STD}}_{\max}}^{\prime }}s_{\mathrm{STD}}^{\prime 3}f_{s^{\prime }}^{\left(\mathrm{STD}\right)}\;ds_{\mathrm{STD}}^{\prime } \end{array}$$

(9c) $$\begin{array}{c} F_{s^{\prime }}:=\frac{\bar{s^{\prime 4}}}{s_{\mathrm{rms}}^{\prime 4}}=\int_{s_{\min}^{\prime }}^{s_{\max}^{\prime }}\frac{s^{\prime 4}\;f_{s^{\prime }}}{s_{\mathrm{rms}}^{\prime 3}}\;d\left(\frac{s^{\prime }}{s_{\mathrm{rms}}^{\prime }}\right)=\int_{s_{{\mathrm{STD}}_{\min}}^{\prime }}^{s_{{\mathrm{STD}}_{\max}}^{\prime }}s_{\mathrm{STD}}^{\prime 4}f_{s^{\prime }}^{\left(\mathrm{STD}\right)}\;ds_{\mathrm{STD}}^{\prime } \end{array}$$

where $\left[s_{\min}^{\prime },s_{\max}^{\prime }\right]$ are the minimal and maximal observed fluctuations, $f_{s^{\prime }}\left(s^{\prime }\right)$ is the probability density function (pdf) for $s^{\prime }$, and $f_{s^{\prime }}^{\left(\mathrm{STD}\right)}$ the corresponding standardised pdf for the standardised variable $s_{\mathrm{STD}}^{\prime }:=s^{\prime }/s_{\mathrm{rms}}^{\prime }$ (8d).

Near the wall, $s^{\prime }$ has positive skewness, decreasing with increasing ${\bar{M}}_{{\mathrm{CL}}_{x}}$ (Figure 8). $S_{s^{\prime }}$ changes sign near $y^{★}\;\;≊\;\;y_{s_{\mathrm{PEAK}}^{\prime }}^{★}$, becoming negative and reaching a negative peak near the beginning of the wake region, as shown in particular by the $Re_{\tau^{★}}\;\;≊\;\;1000$ data (Figure 8). Flatness $F_{s^{\prime }}$ reaches its maximum at approximately the same location. The $Re_{\tau^{★}}\;\;≊\;\;1000$ data indicate a sharp increase in $F_{s^{\prime }}$ near $y^{★}\;\;≊\;\;250$, this region of steep $F_{s^{\prime }}$ increase with $y^{★}$ corresponding to the log-region.

Regarding the ${\bar{M}}_{{\mathrm{CL}}_{x}}$-effect at nearly constant $Re_{\tau^{★}}$ (Figure 8), skewness is shifted towards more negative (or less positive in the near-wall region) values with increasing ${\bar{M}}_{{\mathrm{CL}}_{x}}$ while flatness increases with ${\bar{M}}_{{\mathrm{CL}}_{x}}$ for $y^{★}\;\;⪆\;\;y_{s_{\mathrm{PEAK}}^{\prime }}^{★}$ and decreases for $y^{★}\;\;⪅\;\;y_{s_{\mathrm{PEAK}}^{\prime }}^{★}$.

**Figure 9:**
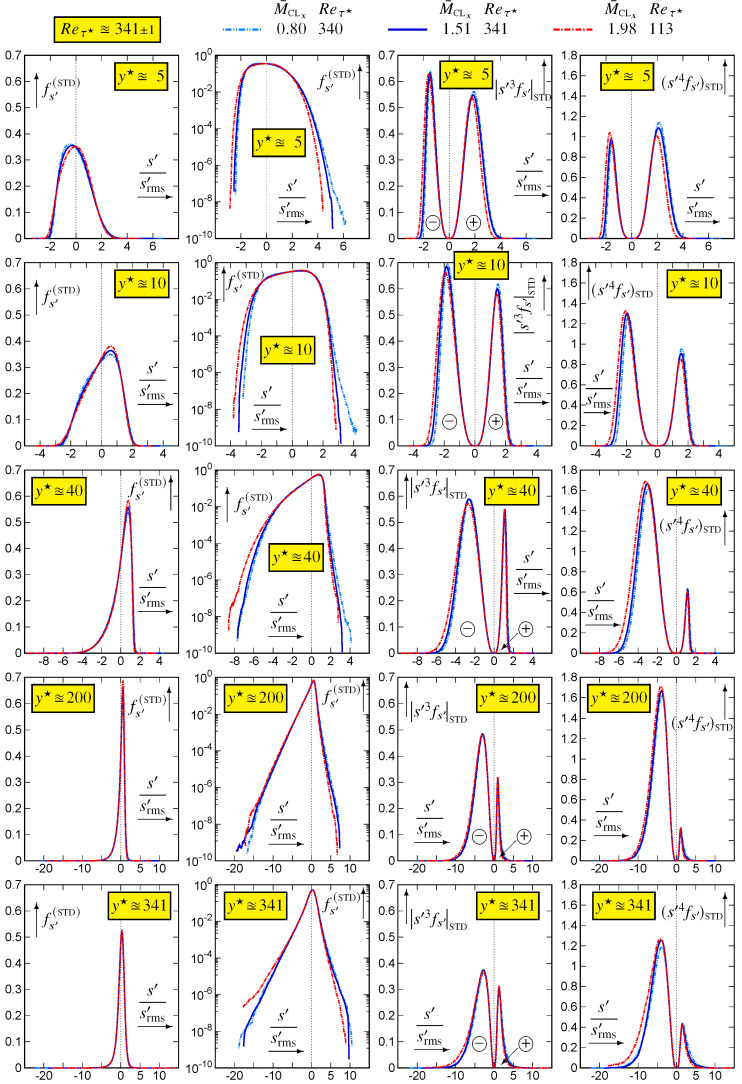
Standardised pdf $f_{s^{\prime }}^{\left(\mathrm{STD}\right)}\;:=\;s_{\mathrm{rms}}^{\prime }f_{s^{\prime }}$ (linear and logscale) and integrands $s_{\mathrm{STD}}^{\prime 3}f_{s^{\prime }}^{\left(\mathrm{STD}\right)}$ and $s_{\mathrm{STD}}^{\prime 4}f_{s^{\prime }}^{\left(\mathrm{STD}\right)}$ of skewness $S_{s^{\prime }}$ ($|s_{\mathrm{STD}}^{\prime 3}|f_{s^{\prime }}^{\left(\mathrm{STD}\right)}$ is plotted with ± indication) and flatness $F_{s^{\prime }}$, plotted against the standardised variable $s_{\mathrm{STD}}^{\prime }\;:=\;s^{\prime }/s_{\mathrm{rms}}^{\prime }$, at different inner-scaled wall distances $y^{★}\;\in \;\left\{5,10,40,200,\delta^{★}≊340\right\}$, for varying ${\bar{M}}_{{\mathrm{CL}}_{x}}\;\in \;\left\{0.80,1.51,1.98\right\}$ at nearly constant $Re_{\tau^{★}}\;\;≊\;\;341$, using dns data [10,11].

Comparison of $f_{s^{\prime }}^{\left(\mathrm{STD}\right)}$ for varying ${\bar{M}}_{{\mathrm{CL}}_{x}}\;\in \;\left\{,1.51,1.98\right\}$ at nearly constant $Re_{\tau^{★}}\;\;≊\;\;341$ (Figure 9) at different $y^{★}$-locations confirms the platykurtic distribution at $y^{★}\;\;≊\;\;10$ where $F_{s^{\prime }}\;\;≊\;\;2$ (Figure 8). With increasing $y^{★}$, $F_{s^{\prime }}$ increases to high values (Figure 8) with increasingly larger range of events in the pdfs (Figure 9) and increasingly negative skewness as can be inferred by comparing the positive and negative parts of the skewness. The increasing probability of occurrence of negative $s^{\prime }$-events, with increasing wall distance, is even more clearly visible in the flatness integrand (Figure 9).

## 6. Conclusions

We used a recently released [10,11] dns database including moments (2- and 3-order) and pdfs (1- and 2-variables) of thermodynamic fluctuations $\left(s^{\prime },p^{\prime },\rho^{\prime },T^{\prime }\right)$ to investigate the fluctuating entropy field in compressible turbulent plane channel flow, and its relation to the other thermodynamic fluctuations. The data analysed in the paper correspond to canonical tpc flow between isothermal walls [5], i.e., to very-cold-wall conditions [11], and the conclusions in the paper apply specifically to this class of flows ($0.58\;\;\leq \;\;-r_{\bar{h}}\;\;\leq \;\;0.65$).

The peak nondimensional fluctuating entropy rms ${\left[s_{\mathrm{rms}}^{\prime }\right]}_{\mathrm{PEAK}}/R_{g}$ depends on both $Re_{\tau^{★}}$ and ${\bar{M}}_{{\mathrm{CL}}_{x}}$, and so does its location in ★-units $y_{s_{\mathrm{PEAK}}^{\prime }}^{★}$. A non-polynomial scaling $s_{\mathrm{rms}}^{\prime }/\left(R_{g}{\bar{M}}_{{\mathrm{CL}}_{x}}^{2}\right)\;\propto \;{\bar{M}}_{{\mathrm{CL}}_{x}}^{\left(0.073{\bar{M}}_{{\mathrm{CL}}_{x}}\right)}$ was used to account for the ${\bar{M}}_{{\mathrm{CL}}_{x}}$-dependence of the peak value. The location $y_{s_{\mathrm{PEAK}}^{\prime }}^{★}$ varies significantly with flow conditions, moving away from the wall with increasing $Re_{\tau^{★}}$ and moving closer to the wall with increasing ${\bar{M}}_{{\mathrm{CL}}_{x}}$.

The joint pdf $f_{s^{\prime }p^{\prime }}$ suggests that $s^{\prime }$ and $p^{\prime }$ are very weakly correlated. This is confirmed by the correlation coefficient $|c_{s^{\prime }p^{\prime }}|\;\;⪅\;\;0.15\;\forall \;y^{★}\;⪆\;5$. In contrast, the joint pdfs $f_{s^{\prime }T^{\prime }}$ and $f_{s^{\prime }\rho^{\prime }}$ reveal a strong correlation (positive for $T^{\prime }$ and negative for $\rho^{\prime }$) in agreement with the corresponding correlation coefficients $c_{s^{\prime }T^{\prime }}\;>\;0$ and $c_{s^{\prime }\rho^{\prime }}\;<\;0$. Moving away from the wall, theses correlations weaken reaching $Re_{\tau^{★}}$-dependent values at centreline. The study of this $Re_{\tau^{★}}$-dependence will be the subject of future work. Examination of the correlation coefficients as y→0 shows that the impact of the strictly isothermal wall condition on the thermodynamic turbulence structure is confined very near the wall ($y^{★}\;<\;2$).

Entropy is slightly positively skewed near the wall ($y^{★}⪅y_{s_{\mathrm{PEAK}}^{\prime }}^{★}$), becoming negatively skewed further away ($y^{★}⪆y_{s_{\mathrm{PEAK}}^{\prime }}^{★}$), to reach a negative-skewness peak at the beginning of the wake-region, where flatness $F_{s^{\prime }}$ reaches its maximum. All these $s^{\prime }$-related quantities $\left(s_{\mathrm{rms}}^{\prime },S_{s^{\prime }},F_{s^{\prime }},c_{s^{\prime }p^{\prime }},c_{s^{\prime }\rho^{\prime }},c_{s^{\prime }T^{\prime }}\right)$ show a $\left(Re_{\tau^{★}},{\bar{M}}_{{\mathrm{CL}}_{x}}\right)$-dependence.

Entropy fluctuations $s^{\prime }$ were expressed as infinite series of powers of $\rho^{\prime }/\bar{\rho }$ and $T^{\prime }/\bar{T}$ (alternatively of $p^{\prime }/\bar{p}$ and $T^{\prime }/\bar{T}$) which were calculated for the general case of variable $c_{p}\left(T\right)$, confirming and generalising previously obtained results that used truncated to two-term series. These general expansions will be used for the study of three-order correlations. Both these analytical results and the dns data highlight the need for a combined study of $s^{\prime }$ statistics with the statistics of the other thermodynamic variables, and this will be the subject of future research.

The higher $Re_{\tau^{★}}\;\;≊\;\;1000$ available data [10,11] are essential to distinguish between near-wall and wake effects (e.g., the $S_{s}^{\prime }$ negative minimum, occurs at the beginning of the wake region), as a separation of inner and outer laws starts to appear. Computations at $\left(Re_{\tau^{★}},{\bar{M}}_{{\mathrm{CL}}_{x}}\right)\;\;≊\;\;\left(1000,2\right)$ are currently ongoing to complete the available ${\bar{M}}_{{\mathrm{CL}}_{x}}\;\in \;\left\{0.8,1.5\right\}$ data. Nonetheless, the need for higher-$Re_{\tau^{★}}$ data is obvious and research efforts should focus on this particularly demanding in computational resources objective.

Finally, data for different thermal wall-conditions are required to investigate the influence of $r_{\bar{h}}$ on entropy fluctuations and on the thermodynamic turbulence structure in general.

## Data Availability

The dns data are available at Gerolymos, G. A.; Vallet, I. (2024), “Compressible turbulent plane channel DNS database”, Mendeley Data, V1, https://data.mendeley.com/datasets/wt8t5kxzbs/1 (accessed on 26 April 2024).

## Abbreviations

The following abbreviations are used in this manuscript:

CC, CCs	correlation coefficients
CV	coefficient of variation
DNS	direct numerical simulation
HCB	Huang–Coleman–Bradshaw [13]
HIT	homogeneous isotropic turbulence
HoTs	higher-order terms
pdf	probability density function
rms	root-mean square
TBL	turbulent boundary layer
TPC	turbulent plane channel
ZPG	zero pressure gradient

## Appendix A. Series Expansions of Entropy

We consider flows for which the equation of state (EoS) is adequately represented by the so-called perfect-gas EoS [26] (pp. 1–38):

(A1a) $$\begin{array}{c} Z:=\frac{p}{\rho R_{g}T}=1\Rightarrow \left\{\begin{array}{c} c_{p}=c_{p}\left(T\right)\\ c_{v}=c_{v}\left(T\right)\\ c_{p}\left(T\right)-c_{v}\left(T\right)=R_{g}=\mathrm{const} \end{array}\right. \end{array}$$

Within this framework of Z=1 thermodynamics (A1a), entropy is defined by either one of the equivalent differential relations [26] (pp. 1–38)

(A1b) $$\begin{array}{cccccccccccccccc} \frac{ds}{R_{g}}\overset{\left(A1a\right)}{=} & \frac{c_{p}\left(T\right)}{R_{g}}\frac{dT}{T}-\frac{dp}{p} & \Rightarrow & \frac{{s|}_{\left(p,T\right)}{-s|}_{\left(\bar{p},\bar{T}\right)}}{R_{g}} & = & \int_{\bar{T}}^{T} & \frac{c_{p}\left(\vartheta \right)}{R_{g}\vartheta }d\vartheta & & - & \ln\left(\frac{p}{\bar{p}}\right) & = & \int_{\bar{T}}^{\bar{T}+T^{\prime }} & \frac{c_{p}\left(\vartheta \right)}{R_{g}\vartheta }d\vartheta & & - & \ln\left(1+\frac{p^{\prime }}{\bar{p}}\right) \end{array}$$

(A1c) $$\begin{array}{cccccccccccccccc} \frac{ds}{R_{g}}\overset{\left(A1a\right)}{=} & \frac{c_{v}\left(T\right)}{R_{g}}\frac{dT}{T}-\frac{d\rho }{\rho } & \Rightarrow & \frac{{s|}_{\left(\rho ,T\right)}{-s|}_{\left(\bar{\rho },\bar{T}\right)}}{R_{g}} & = & \int_{\bar{T}}^{T} & \frac{c_{v}\left(\vartheta \right)}{R_{g}\vartheta }d\vartheta & & - & \ln\left(\frac{\rho }{\bar{\rho }}\right) & = & \int_{\bar{T}}^{\bar{T}+T^{\prime }} & \frac{c_{v}\left(\vartheta \right)}{R_{g}\vartheta }d\vartheta & & - & \ln\left(1+\frac{\rho^{\prime }}{\bar{\rho }}\right) \end{array}$$

providing the difference between the instantaneous entropy $s\;=\;\bar{s}+s^{\prime }$ at the corresponding flow conditions ($p=\bar{p}+p^{\prime }$, $\rho =\bar{\rho }+\rho^{\prime }$, $T=\bar{T}+T^{\prime }$) and the entropy corresponding to mean flow conditions. Notice that because of the EoS (A1a) nonlinearity, implying $\bar{p}\neq \bar{\rho }R_{g}\bar{T}$, the two reference states in (A1) are slightly different (${s|}_{\left(\bar{p},\bar{T}\right)}{\;\neq \;s|}_{\left(\bar{\rho },\bar{T}\right)}$), whereas the nonlinearity of (A1) implies that they are also slightly different from the mean entropy $\bar{s}$. These $O({\mathrm{CV}}_{\rho^{\prime }}^{2},{\mathrm{CV}}_{T^{\prime }}^{2},{\mathrm{CV}}_{p^{\prime }}^{2})$ small differences are calculated in Appendix A.5.

In TPCs and ZPG-TBLs, the extreme instantaneous levels of $|p^{\prime }/\bar{p}|$, $|\rho^{\prime }/\bar{\rho }|$ and $|T^{\prime }/\bar{T}|$ are <1 so that (A1) can be expanded into convergent infinite series of these instantaneous relative levels $\left\{p^{\prime }/\bar{p},\rho^{\prime }/\bar{\rho },T^{\prime }/\bar{T}\right\}$.

### Appendix A.1. Basic Expansions

The following basic expansions will be used in the calculations

(A2a) $$\begin{array}{cc} \ln(1+x)= & \overset{x\;-\;\frac{1}{2}x^{2}\;+\;\frac{1}{3}x^{3}\;+\;\cdots }{\overset{︷}{\sum_{\ell =1}^{\infty }\frac{{\left(-1\right)}^{\ell +1}}{\ell }x^{\ell }}}=\overset{{\mathrm{TJ}}_{n}\left[\ln\left(1+x\right);x;0\right]}{\overset{︷}{\sum_{\ell =1}^{n}\frac{{\left(-1\right)}^{\ell +1}}{\ell }x^{\ell }}}+\overset{O(x^{n+1})}{\overset{︷}{\sum_{\ell =n+1}^{\infty }\frac{{\left(-1\right)}^{\ell +1}}{\ell }x^{\ell }}}\;\;\forall \left|x\right|\;<\;1 \end{array}$$

(A2b) $$\begin{array}{cc} \int \frac{x^{n}dx}{1+x}= & {\left(-1\right)}^{n}\left[\ln\left(1+x\right)-\underset{{\mathrm{TJ}}_{n}\left[\ln\left(1+x\right);x;0\right]}{\underset{︸}{\sum_{\ell =1}^{n}\frac{{\left(-1\right)}^{\ell +1}}{\ell }x^{\ell }}}\right]\;\;\forall \;n\;\in \;N_{>0}\;\forall x\;>\;-1 \end{array}$$

(A2c) $$\begin{array}{cc} \int \frac{x^{n}dx}{1+x}= & {\left(-1\right)}^{n}\sum_{\ell =n+1}^{\infty }\frac{{\left(-1\right)}^{\ell +1}}{\ell }x^{\ell }\;\;\forall \left|x\right|\;<\;1 \end{array}$$

where ${\mathrm{TJ}}_{n}\left[\ln\left(1+x\right);x;0\right]$ (A2a) denotes the n-jet [27] (Section 3.6, pp. 43–46), i.e., the truncated Taylor series of ln(1+x) in the neighbourhood of x=0, including terms up to $O(x^{n})$.

**Proof of** **(A2).** (A2a) is the standard Taylor expansion of ln(1+x)[28] (p. 140) in the neighbourhood of x=0, its general expression obtained by straightforward induction for the derivative ${\left[\ln\left(1+x\right)\right]}^{\left(\ell \right)}$. (A2b) is obtained by induction from the recurrence relation

$$\begin{array}{c} \int \frac{x^{n}dx}{1+x}=\int \frac{x^{n}+x^{n-1}-x^{n-1}}{1+x}dx=\int x^{n-1}dx-\int \frac{x^{n-1}dx}{1+x}=\frac{x^{n}}{n}-\int \frac{x^{n-1}dx}{1+x} \end{array}$$

starting with n=1 and using

$$\begin{array}{c} \int \frac{dx}{1+x}=\ln\left(1+x\right) \end{array}$$

The finite series in (A2b) are precisely the Taylor-jet ${\mathrm{TJ}}_{n}\left[\ln\left(1+x\right);x;0\right]$ defined in (A2a), so that replacing the expansion (A2a) for ln(1+x) in (A2b) yields (A2c). The alternating series in (A2a, A2c) are obviously convergent ∀|x|<1 [28] (p. 130).  □

### Appendix A.2. The c_p_ (T)-Integral

For the simple case $c_{p}\;=\;\mathrm{const}$, which is widely used in dns computations, the integration of (A1b, A1c) is of course straightforward. However in high-Mach-number flows, where large temperature variations occur, variable $c_{p}\left(T\right)$ [29,30] thermodynamics must be used.

In the general case of variable-$c_{p}\left(T\right)$ thermodynamics, the $c_{p}$ (A1b) or $c_{v}$ (A1c) integrals must be calculated using the Taylor expansions of $c_{p}\left(T\right)$. The Taylor series of $c_{p}\left(T\right)\;=\;c_{p}\left(\bar{T}+T^{\prime }\right)$ around $\bar{T}$ read [28] (pp. 137–138)

(A3a) $$\begin{array}{c} c_{p}\left(\bar{T}+T^{\prime }\right)=c_{p}\left(\bar{T}\right)+\sum_{k=1}^{\infty }\left(\frac{1}{k!}{\left.\frac{d^{k}c_{p}}{dT^{k}}\right|}_{\bar{T}}\;{\left(T^{\prime }\right)}^{\;k}\right)=c_{p}\left(\bar{T}\right)+\sum_{k=1}^{\infty }\left(\frac{{\bar{T}}^{k}}{k!}{\left.\frac{d^{k}c_{p}}{dT^{k}}\right|}_{\bar{T}}\;{\left(\frac{T^{\prime }}{\bar{T}}\right)}^{\;\;\;k}\right) \end{array}$$

and equivalently,

(A3b) $$\begin{array}{c} \zeta :=\frac{\vartheta -\bar{T}}{\bar{T}}\Leftrightarrow \vartheta =\bar{T}\left(1+\zeta \right)\overset{\left(A3a\right)}{\Rightarrow }c_{p}\left(\vartheta \right)=c_{p}\left(\bar{T}\right)+\sum_{k=1}^{\infty }\frac{{\bar{T}}^{k}}{k!}{\left.\frac{d^{k}c_{p}}{dT^{k}}\right|}_{\bar{T}}\;\zeta^{k} \end{array}$$

which can be used to expand the integral in (A1b):

(A3c) $$\begin{array}{cc} & \int_{\bar{T}}^{\bar{T}+T^{\prime }}\frac{c_{p}\left(\vartheta \right)}{\vartheta }d\vartheta \overset{\left(A3b\right)}{=}\int_{\zeta =0}^{\zeta =\frac{T^{\prime }}{\bar{T}}}\frac{c_{p}\left(\bar{T}\;\left(1+\zeta \right)\right)}{\bar{T}\;\left(1+\zeta \right)}\underset{d\vartheta }{\underset{︸}{d\left(\bar{T}\;\left(1+\zeta \right)\right)}}=\int_{0}^{\frac{T^{\prime }}{\bar{T}}}\frac{c_{p}\left(\bar{T}\;\left(1+\zeta \right)\right)}{1+\zeta }d\zeta \\ \overset{\left(A3b\right)}{=} & \int_{0}^{\frac{T^{\prime }}{\bar{T}}}\left[c_{p}\left(\bar{T}\right)+\sum_{k=1}^{\infty }\left(\frac{{\bar{T}}^{k}}{k!}{\left.\frac{d^{k}c_{p}}{dT^{k}}\right|}_{\bar{T}}\;\zeta^{k}\right)\right]\frac{d\zeta }{1+\zeta }=c_{p}\left(\bar{T}\right)\;\int_{0}^{\frac{T^{\prime }}{\bar{T}}}\frac{d\zeta }{1+\zeta }+\sum_{k=1}^{\infty }\left[\frac{{\bar{T}}^{k}}{k!}{\left.\frac{d^{k}c_{p}}{dT^{k}}\right|}_{\bar{T}}\;\int_{0}^{\frac{T^{\prime }}{\bar{T}}}\frac{\zeta^{k}d\zeta }{1+\zeta }\right]\\ \overset{\left(A2\right)}{=} & c_{p}\left(\bar{T}\right)\;\ln\left(1\;+\;\frac{T^{\prime }}{\bar{T}}\right)+\sum_{k=1}^{\infty }\left(\frac{{\bar{T}}^{k}}{k!}{\left.\frac{d^{k}c_{p}}{dT^{k}}\right|}_{\bar{T}}\;{\left(-1\right)}^{k}\sum_{\ell =k+1}^{\infty }\left[\frac{{\left(-1\right)}^{\ell +1}}{\ell }{\left(\frac{T^{\prime }}{\bar{T}}\right)}^{\;\;\;\ell }\right]\right)\\ = & c_{p}\left(\bar{T}\right)\;\ln\left(1+\frac{T^{\prime }}{\bar{T}}\right)+\sum_{k=1}^{\infty }\sum_{\ell =k+1}^{\infty }\left[\frac{{\bar{T}}^{k}}{k!}{\left.\frac{d^{k}c_{p}}{dT^{k}}\right|}_{\bar{T}}\frac{{\left(-1\right)}^{k+\ell +1}}{\ell }{\left(\frac{T^{\prime }}{\bar{T}}\right)}^{\;\;\;\ell }\right]\\ = & c_{p}\left(\bar{T}\right)\;\ln\left(1+\frac{T^{\prime }}{\bar{T}}\right)+\sum_{\ell =2}^{\infty }\left[\left(\sum_{k=1}^{\ell -1}\frac{{\bar{T}}^{k}}{k!}{\left.\frac{d^{k}c_{p}}{dT^{k}}\right|}_{\bar{T}}\frac{{\left(-1\right)}^{k+\ell +1}}{\ell }\right){\left(\frac{T^{\prime }}{\bar{T}}\right)}^{\;\;\;\ell }\right] \end{array}$$

where the positivity of absolute temperature $T=\bar{T}+T^{\prime }>0\Leftrightarrow 1+T^{\prime }/\bar{T}>0$ ensures the existence of the logarithm in (A3c). Furthermore, the condition $|T^{\prime }|\leq \bar{T}$ is invariably satisfied in practical turbulent aerodynamic flows. Notice that the identity [31,32]

$$\begin{array}{c} \begin{array}{ccc} 1\leq & k & <\infty \\ k+1\leq & \ell & <\infty \end{array}\Leftrightarrow \begin{array}{ccc} 1\leq & k & <\infty \\ k+2\leq & \ell +k & <\infty \\ k+1\leq & \ell & <\infty \end{array}\Leftrightarrow \begin{array}{ccc} 1\leq & k & <\infty \\ 2\leq & \ell & <\infty \\ k\leq & \ell -1 & <\infty \end{array}\Leftrightarrow \begin{array}{ccc} 2\leq & \ell & <\infty \\ 1\leq & k & \leq \ell -1 \end{array} \end{array}$$

was used to rearrange the double summation limits in (A3c) into factors of increasing powers of ${\left(T^{\prime }/\bar{T}\right)}^{\ell }$. Finally, injecting the expansion (A2a) of $\ln(1+T^{\prime }/\bar{T})$ around $T^{\prime }/\bar{T}=0$ in (A3c) gives the final expressions for the $c_{p}\left(T\right)$-integral (A1b):

(A4) $$\begin{array}{c} \int_{\bar{T}}^{\bar{T}+T^{\prime }}\frac{c_{p}\left(\vartheta \right)}{\vartheta }d\vartheta \overset{\left(A3c,A2a\right)}{=}c_{p}\left(\bar{T}\right)\;\frac{T^{\prime }}{\bar{T}}+\sum_{\ell =2}^{\infty }\left[\frac{{\left(-1\right)}^{\ell +1}}{\ell }{\left(\frac{T^{\prime }}{\bar{T}}\right)}^{\;\;\;\ell }\;c_{p}\left(\bar{T}\right)\left(1+\sum_{k=1}^{\ell -1}\frac{{\left(-1\right)}^{k}}{k!}\frac{{\bar{T}}^{k}}{c_{p}\left(\bar{T}\right)}{\left.\frac{d^{k}c_{p}}{dT^{k}}\right|}_{\bar{T}}\right)\right] \end{array}$$

Notice that the variation of $c_{p}$ with temperature does not affect the linear term (A4), since the leading term of $c_{p}$-derivatives is quadratic in the instantaneous fluctuation intensity (A3c). Using the condition ℓ>1 in the summation symbol substack (the sum is considered =0 when ℓ=1) expression (A4) is cast in a more concise expression:

(A5) $$\begin{array}{c} \int_{\bar{T}}^{\bar{T}+T^{\prime }}\frac{c_{p}\left(\vartheta \right)}{\vartheta }d\vartheta \overset{\left(A4\right)}{=}\sum_{\ell =1}^{\infty }\left[\frac{{\left(-1\right)}^{\ell +1}}{\ell }{\left(\frac{T^{\prime }}{\bar{T}}\right)}^{\;\;\;\ell }\;c_{p}\left(\bar{T}\right)\left(1+\sum_{\begin{array}{c} k=1\\ \ell >1 \end{array}}^{\ell -1}\frac{{\left(-1\right)}^{k}}{k!}\frac{{\bar{T}}^{k}}{c_{p}\left(\bar{T}\right)}{\left.\frac{d^{k}c_{p}}{dT^{k}}\right|}_{\bar{T}}\right)\right] \end{array}$$

### Appendix A.3. Expansion of s(p,T)

Using (A5) for the $c_{p}\left(T\right)$-integral and the Taylor expansion (A2a) of $\ln(1\;+\;p^{\prime }/\bar{p})$ around $p^{\prime }/\bar{p}\;=\;0$ yields the expansion of (A1b):

(A6a) $$\begin{array}{c} \frac{{s-s|}_{\left(\bar{p},\bar{T}\right)}}{R_{g}}\overset{\left(A1b,A5,A2a\right)}{=}\sum_{\ell =1}^{\infty }\left(\frac{{\left(-1\right)}^{\ell +1}}{\ell }\left[\;-\;\;{\left(\frac{p^{\prime }}{\bar{p}}\right)}^{\;\;\;\ell }\;+\;\;{\left(\frac{T^{\prime }}{\bar{T}}\right)}^{\;\;\;\ell }\;\frac{c_{p}\left(\bar{T}\right)}{R_{g}}\left(1+\sum_{\begin{array}{c} k=1\\ \ell >1 \end{array}}^{\ell -1}\frac{{\left(-1\right)}^{k}}{k!}\frac{{\bar{T}}^{k}}{c_{p}\left(\bar{T}\right)}{\left.\frac{d^{k}c_{p}}{dT^{k}}\right|}_{\bar{T}}\right)\right]\right) \end{array}$$

Averaging (A6a) highlights the small difference between the Reynolds-averaged entropy $\bar{s}$ and the entropy ${s|}_{\left(\bar{p},\bar{T}\right)}$ corresponding to the state defined by the couple $\left(\bar{p},\bar{T}\right)$:

(A6b) $$\begin{array}{c} \frac{\bar{s}{-s|}_{\left(\bar{p},\bar{T}\right)}}{R_{g}}\overset{\left(A6a\right)}{=}\underset{O({\mathrm{CV}}_{p^{\prime }}^{2},{\mathrm{CV}}_{T^{\prime }}^{2})}{\underset{︸}{\sum_{\ell =2}^{\infty }\left(\frac{{\left(-1\right)}^{\ell +1}}{\ell }\left[\;-\;\;\bar{{\left(\frac{p^{\prime }}{\bar{p}}\right)}^{\;\;\;\ell }}\;+\;\bar{{\left(\frac{T^{\prime }}{\bar{T}}\right)}^{\;\;\;\ell }}\;\frac{c_{p}\left(\bar{T}\right)}{R_{g}}\left(1+\sum_{k=1}^{\ell -1}\frac{{\left(-1\right)}^{k}}{k!}\frac{{\bar{T}}^{k}}{c_{p}\left(\bar{T}\right)}{\left.\frac{d^{k}c_{p}}{dT^{k}}\right|}_{\bar{T}}\right)\right]\right)}} \end{array}$$

Replacing $s\;=\;\bar{s}\;+\;s^{\prime }$ in (A6a), we obtain the expansion for the entropy fluctuations in terms of the relative instantaneous intensities $\left(p^{\prime }/\bar{p},T^{\prime }/\bar{T}\right)$ of pressure and temperature fluctuations:

(A6c) $$\begin{array}{c} \frac{s^{\prime }}{R_{g}}\overset{\left(A6a\right)}{=}\frac{{s|}_{\left(\bar{p},\bar{T}\right)}-\bar{s}}{R_{g}}+\sum_{\ell =1}^{\infty }\left(\frac{{\left(-1\right)}^{\ell +1}}{\ell }\left[\;-\;\;{\left(\frac{p^{\prime }}{\bar{p}}\right)}^{\;\;\;\ell }\;+\;{\left(\frac{T^{\prime }}{\bar{T}}\right)}^{\;\;\;\ell }\;\frac{c_{p}\left(\bar{T}\right)}{R_{g}}\left(1\;+\;\sum_{\begin{array}{c} k=1\\ \ell >1 \end{array}}^{\ell -1}\frac{{\left(-1\right)}^{k}}{k!}\frac{{\bar{T}}^{k}}{c_{p}\left(\bar{T}\right)}{\left.\frac{d^{k}c_{p}}{dT^{k}}\right|}_{\bar{T}}\right)\right]\right) \end{array}$$

which contain the small nonfluctuating term $\bar{s}{-s|}_{\left(\bar{p},\bar{T}\right)}$ (A6b).

### Appendix A.4. Expansion of s(ρ,T)

Notice first that the Z=1 EoS (A1a) implies [26] (pp. 1–38)

(A7) $$\begin{array}{c} \left(A1a\right)\Rightarrow c_{v}\left(T\right)=c_{p}\left(T\right)-R_{g}\Rightarrow \frac{d^{n}c_{v}}{dT^{n}}=\frac{d^{n}c_{p}}{dT^{n}}\;\forall \;n\;\in \;N_{>0} \end{array}$$

so that Taylor expansions of $c_{v}\left(T\right)$ and $c_{p}\left(T\right)$ are identical except for the constant term. Obviously, the $c_{v}\left(T\right)$-integral in (A1c) can be expanded in exactly the same way as the $c_{p}\left(T\right)$-integral in (A1b), i.e., replacing $c_{p}$ by $c_{v}$ in (A5)

(A8) $$\begin{array}{c} \int_{\bar{T}}^{\bar{T}+T^{\prime }}\frac{c_{v}\left(\vartheta \right)}{\vartheta }d\vartheta \overset{\left(A5\right)}{=}\sum_{\ell =1}^{\infty }\left[\frac{{\left(-1\right)}^{\ell +1}}{\ell }{\left(\frac{T^{\prime }}{\bar{T}}\right)}^{\;\;\;\ell }\;c_{v}\left(\bar{T}\right)\left(1+\sum_{\begin{array}{c} k=1\\ \ell >1 \end{array}}^{\ell -1}\frac{{\left(-1\right)}^{k}}{k!}\frac{{\bar{T}}^{k}}{c_{v}\left(\bar{T}\right)}{\left.\frac{d^{k}c_{v}}{dT^{k}}\right|}_{\bar{T}}\right)\right] \end{array}$$

and the expansion of (A1c) is obtained by expanding $\ln(1\;+\;\rho^{\prime }/\bar{\rho })$ around $\rho^{\prime }/\bar{\rho }\;=\;0$ (A2a):

(A9a) $$\begin{array}{c} \frac{{s-s|}_{\left(\bar{\rho },\bar{T}\right)}}{R_{g}}\overset{\left(A1c,A8,A2a\right)}{=}\sum_{\ell =1}^{\infty }\left(\frac{{\left(-1\right)}^{\ell +1}}{\ell }\left[\;-\;\;{\left(\frac{\rho^{\prime }}{\bar{\rho }}\right)}^{\;\;\;\ell }\;+\;{\left(\frac{T^{\prime }}{\bar{T}}\right)}^{\;\;\;\ell }\;\frac{c_{v}\left(\bar{T}\right)}{R_{g}}\left(1+\sum_{\begin{array}{c} k=1\\ \ell >1 \end{array}}^{\ell -1}\frac{{\left(-1\right)}^{k}}{k!}\frac{{\bar{T}}^{k}}{c_{v}\left(\bar{T}\right)}{\left.\frac{d^{k}c_{v}}{dT^{k}}\right|}_{\bar{T}}\right)\right]\right) \end{array}$$

Averaging (A9a) quantifies the small difference between the Reynolds-averaged entropy $\bar{s}$ and the entropy ${s|}_{\left(\bar{\rho },\bar{T}\right)}$ corresponding to the state defined by the couple $\left(\bar{\rho },\bar{T}\right)$

(A9b) $$\begin{array}{c} \frac{\bar{s}{-s|}_{\left(\bar{\rho },\bar{T}\right)}}{R_{g}}\overset{\left(A9a\right)}{=}\underset{O({\mathrm{CV}}_{\rho^{\prime }}^{2},{\mathrm{CV}}_{T^{\prime }}^{2})}{\underset{︸}{\sum_{\ell =2}^{\infty }\left(\frac{{\left(-1\right)}^{\ell +1}}{\ell }\left[\;-\;\;\bar{{\left(\frac{\rho^{\prime }}{\bar{\rho }}\right)}^{\;\;\;\ell }}\;+\;\bar{{\left(\frac{T^{\prime }}{\bar{T}}\right)}^{\;\;\;\ell }}\;\frac{c_{v}\left(\bar{T}\right)}{R_{g}}\left(1+\sum_{k=1}^{\ell -1}\frac{{\left(-1\right)}^{k}}{k!}\frac{{\bar{T}}^{k}}{c_{v}\left(\bar{T}\right)}{\left.\frac{d^{k}c_{v}}{dT^{k}}\right|}_{\bar{T}}\right)\right]\right)}} \end{array}$$

Replacing $s\;=\;\bar{s}\;+\;s^{\prime }$ in (A9a) yields the expansion for the entropy fluctuations in terms of the relative instantaneous intensities $\left(\rho^{\prime }/\bar{\rho },T^{\prime }/\bar{T}\right)$ of density and temperature fluctuations:

(A9c) $$\begin{array}{c} \frac{s^{\prime }}{R_{g}}\overset{\left(A9a\right)}{=}\frac{{s|}_{\left(\bar{\rho },\bar{T}\right)}-\bar{s}}{R_{g}}+\sum_{\ell =1}^{\infty }\left(\frac{{\left(-1\right)}^{\ell +1}}{\ell }\left[\;-\;\;{\left(\frac{\rho^{\prime }}{\bar{\rho }}\right)}^{\;\;\;\ell }\;+\;{\left(\frac{T^{\prime }}{\bar{T}}\right)}^{\;\;\;\ell }\;\frac{c_{v}\left(\bar{T}\right)}{R_{g}}\left(1+\sum_{\begin{array}{c} k=1\\ \ell >1 \end{array}}^{\ell -1}\frac{{\left(-1\right)}^{k}}{k!}\frac{{\bar{T}}^{k}}{c_{v}\left(\bar{T}\right)}{\left.\frac{d^{k}c_{v}}{dT^{k}}\right|}_{\bar{T}}\right)\right]\right) \end{array}$$

### Appendix A.5. Mean Entropy $\bar{s}$ vs. Mean-State Entropies $s{|}_{\left(\bar{p},\bar{T}\right)}$, and $s{|}_{\left(\bar{\rho },\bar{T}\right)}$

There is a slight difference in the reference state between (A6, A9) since the EoS (A1a) implies $\bar{p}\;∼\;\bar{\rho }R_{g}\bar{T}\;+\;O\left({\mathrm{CV}}_{\rho^{\prime }}{\mathrm{CV}}_{T^{\prime }}\right)$, i.e., the pressure defined by the couple $\left(\bar{\rho },\bar{T}\right)$ is not exactly $\bar{p}$, the difference being formally $O({\mathrm{CV}}_{\rho^{\prime }}{\mathrm{CV}}_{T^{\prime }})$ [20] ((2.4), p. 452). It is noteworthy that subtracting (A6b) from (A9b), and using $c_{p}\left(T\right)\;-\;c_{v}\left(T\right)\;=\;R_{g}$ (A1a), which implies the equality of derivatives (A7), yields the difference between the two reference-state entropies

(A10) $$\begin{array}{c} \frac{{s|}_{\left(\bar{p},\bar{T}\right)}{-s|}_{\left(\bar{\rho },\bar{T}\right)}}{R_{g}}\overset{\left(A6b,A9b,A1a,A7\right)}{=}\sum_{\ell =2}^{\infty }\left(\frac{{\left(-1\right)}^{\ell }}{\ell }\left[\bar{{\left(\frac{\rho^{\prime }}{\bar{\rho }}\right)}^{\;\;\;\ell }}+\bar{{\left(\frac{T^{\prime }}{\bar{T}}\right)}^{\;\;\;\ell }}\;-\bar{{\left(\frac{p^{\prime }}{\bar{p}}\right)}^{\;\;\;\ell }}\right)]\right)∼\frac{1}{2}\left({\mathrm{CV}}_{\rho^{\prime }}^{2}+{\mathrm{CV}}_{T^{\prime }}^{2}-{\mathrm{CV}}_{p^{\prime }}^{2}\right)+\mathrm{HoTs} \end{array}$$

which depends exclusively on the statistics of $\left\{p^{\prime },\rho^{\prime },T^{\prime }\right\}$ and is independent of the derivatives of $c_{p}\left(T\right)$.
